# Intelligent Soft Opto‐Magnetic Robot for Minimally Invasive Interventional Therapy

**DOI:** 10.1002/advs.202520344

**Published:** 2026-01-21

**Authors:** Jingjing Guo, Xiaoyan Guo, Jian Wei, Miaowen Jiang, Qifa Su, Xian Xia, Zhuozhou Li, Rong Cai, Jianguo Ma, Bo Fu, Ming Li, Jing Zhong, Lijun Xu, Xunming Ji

**Affiliations:** ^1^ School of Instrumentation and Optoelectronic Engineering Beihang University Beijing China; ^2^ Hangzhou International Innovation Institute Beihang University Hangzhou China; ^3^ Department of Interventional Radiology Beijing Friendship Hospital Capital Medical University Beijing China; ^4^ Beijing Institute of Brain Disorders Capital Medical University Beijing China; ^5^ School of Engineering Medicine Beihang University Beijing China; ^6^ China‐America Institute of Neuroscience and Beijing Institute of Geriatrics Xuanwu Hospital Capital Medical University Beijing China; ^7^ Department of Neurology and Neurosurgery Xuanwu Hospital Capital Medical University Beijing China

**Keywords:** minimally invasive interventions, optical tactile sensor, photothermal ablation therapy, soft robotics

## Abstract

Minimally invasive interventions (MII) have transformed surgical practice by reducing patient trauma, speeding recovery, and improving patient outcomes. However, their effectiveness remains constrained by the lack of tactile feedback in confined spaces, reliance on manual dexterity, and dependence on radiation‐based imaging. Remotely controlled miniature soft robots that integrate tactile sensing, real‐time monitoring, and therapeutic delivery offer a promising route to overcome these barriers, but achieving such multifunctionality within a single device has yet to be realized. Here, we introduce an intelligent soft opto‐magnetic (iSOM) robot that integrates magnetic actuation, skin‐mimic tactile sensing, and photothermal ablation in a millimeter‐scale design. The robot comprises a compliant polymer sheath configured with optical pathways for sensing and therapeutic light delivery, a magnetically steerable body for controlled navigation, and a tissue‐conforming tip designed to provide tactile feedback and localized photothermal therapy. Driven by magnetic fields and laser excitation, the robot can actively navigate tortuous anatomy to perform targeted ablation therapy, while simultaneously providing real‐time feedback on both navigation and treatment to ensure precision and safety. Its capabilities were validated in proof‐of‐concept demonstrations, including real‐time tracked photothermal ablation in porcine tissue, targeted cardiac ablation in cardiovascular phantoms, and image‐guided therapeutic interventions in ex vivo and in vivo anatomical systems, underscoring its translational potential for high‐precision MII.

## Graphical abstract

An intelligent soft opto‐magnetic (iSOM) robot is developed that integrates remote magnetic actuation, skin‐mimic tactile perception, and photothermal ablation in a near‐millimeter‐scale design. It enables precise intraluminal navigation and localized ablation while providing real‐time optical feedback to support safe and accurate minimally invasive interventions.

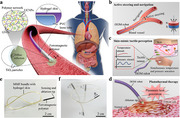

## 1 Introduction

Minimally invasive interventions (MII), utilizing long instruments inserted through natural orifices or small skin incisions, are pivotal in the diagnosis and treatment of various human diseases [1, 2]. These techniques enable precise, image‐guided catheter delivery to targeted lesions while minimizing patient trauma, offering superior therapeutic outcomes compared to conventional treatments [3]. Thermal ablation therapy (TAT), in particular, has gained significant attention in recent years for its minimally invasive application in treating cancers, cardiovascular, gastrointestinal, and urological disorders, with the benefits of low cost, rapid recovery, and reduced postoperative complications [4, 5, 6]. TAT harnesses thermal energy sources (e.g., microwave [7, 8], radiofrequency [9, 10], or laser [11, 12, 13]) to induce localized hyperthermia, elevating tissue temperatures above 50°C, resulting in irreversible coagulative necrosis and effective ablation of diseased tissue. Despite its widespread clinical applications, TAT demands a high level of precision due to the risk of damaging surrounding healthy tissues, and the precision required for successful ablation is highly operator‐dependent, making it susceptible to fatigue and human error [14]. Moreover, the loss of tactile feedback during minimally invasive procedures limits the surgeon's ability to accurately assess the tissue being treated, potentially leading to inadvertent injuries [15, 16]. Additionally, the reliance on imaging modalities such as X‐rays or CT scans for real‐time guidance during ablation introduces risks associated with radiation exposure for both surgeons and patients [17].

Recent advances in soft robotics are poised to revolutionize TAT by enabling remote‐controlled interventions [18, 19, 20]. The integration of thermal ablation catheters with small‐scale soft robots enhances surgical precision, controllability, and safety by allowing navigation through hard‐to‐reach areas of the body, while eliminating radiation exposure to the surgeons during real‐time imaging. Magnetic fields, recognized for their safety and deep tissue penetration, are promising sources of energy for remotely actuating these surgical robots [21, 22, 23, 24]. For instance, Kim et al. designed a soft magnetic robot using ferromagnetic nanocomposites, capable of omnidirectional steering within intricate brain vasculature upon magnetic actuation [23]. The magnetic fields needed to actuate such robots can be generated by electromagnets, permanent magnets, or MRI (magnetic resonance imaging) systems. Among these, MRI is particularly ideal, as it can provide high‐resolution imaging without ionizing radiation, enabling both remote guidance and precise localization [25, 26]. However, conventional thermal ablation probes, typically involve the use of metallic components, which are incompatible with the MRI environments. In addition, incorporating tactile feedback during navigation and real‐time monitoring of key parameters during ablation is crucial for reducing surgical risks. Despite clear clinical need, the integration of dynamic monitoring, tactile perception, and ablation therapy into a millimeter‐scale robot that allows active steering and remote control, while ensuring the necessary softness, flexibility, and biocompatibility for minimally invasive procedures, remains a significant and unresolved challenge.

Here, we report an intelligent soft opto‐magnetic (iSOM) robot that seamlessly integrates skin‐mimic thermomechanical sensing, photothermal ablation, and magnetic actuation within a millimeter‐scale soft architecture. Driven by magnetic fields and laser excitation, the iSOM robot enables precise remote navigation through confined anatomical spaces for minimally invasive TAT, while simultaneously providing real‐time feedback to ensure procedural safety and accuracy. The iSOM robot is constructed with a compliant polymer sheath embedding bundled multimodal optical fibers (MMFs) for sensing and therapeutic light delivery, and a magnetic‐responsive distal segment that enables omnidirectional steering under external magnetic fields. The distal tip is engineered with a hemispherical optical structure made from polydimethylsiloxane (PDMS) elastomers integrated with lanthanide‐based upconversion nanoparticles (Ln‐UCNPs) and gold nanoparticles (GNPs), featuring skin‐like softness, deformability, and multimodal sensory functions. Upon near‐infrared (NIR) excitation, the Ln‐UCNPs generate multiband upconversion luminescence (UCL) emissions that spectrally overlap with the resonance of the GNPs, thereby facilitating the excitation of localized surface plasmons. This plasmon‐upconversion coupling mechanism enables spectrally decoupled optical readouts of temperature and pressure, providing skin‐like haptic feedback. Simultaneously, the excited localized surface plasmon resonance (LSPR) enhances photothermal conversion, efficiently transforming light into localized heat for targeted TAT. We thoroughly investigate the optical sensing, magnetic actuation, and photothermal performances of the robot, and as a proof of concept, demonstrate its capabilities for real‐time tracked photothermal ablation therapy in porcine tissue via percutaneous insertion, targeted cardiac ablation in cardiovascular phantoms, and image‐guided therapeutic interventions in ex vivo and in vivo anatomical systems.

## 2 Results and Discussion

### 2.1 Design and Fabrication of the iSOM Robot

**FIGURE 1:**
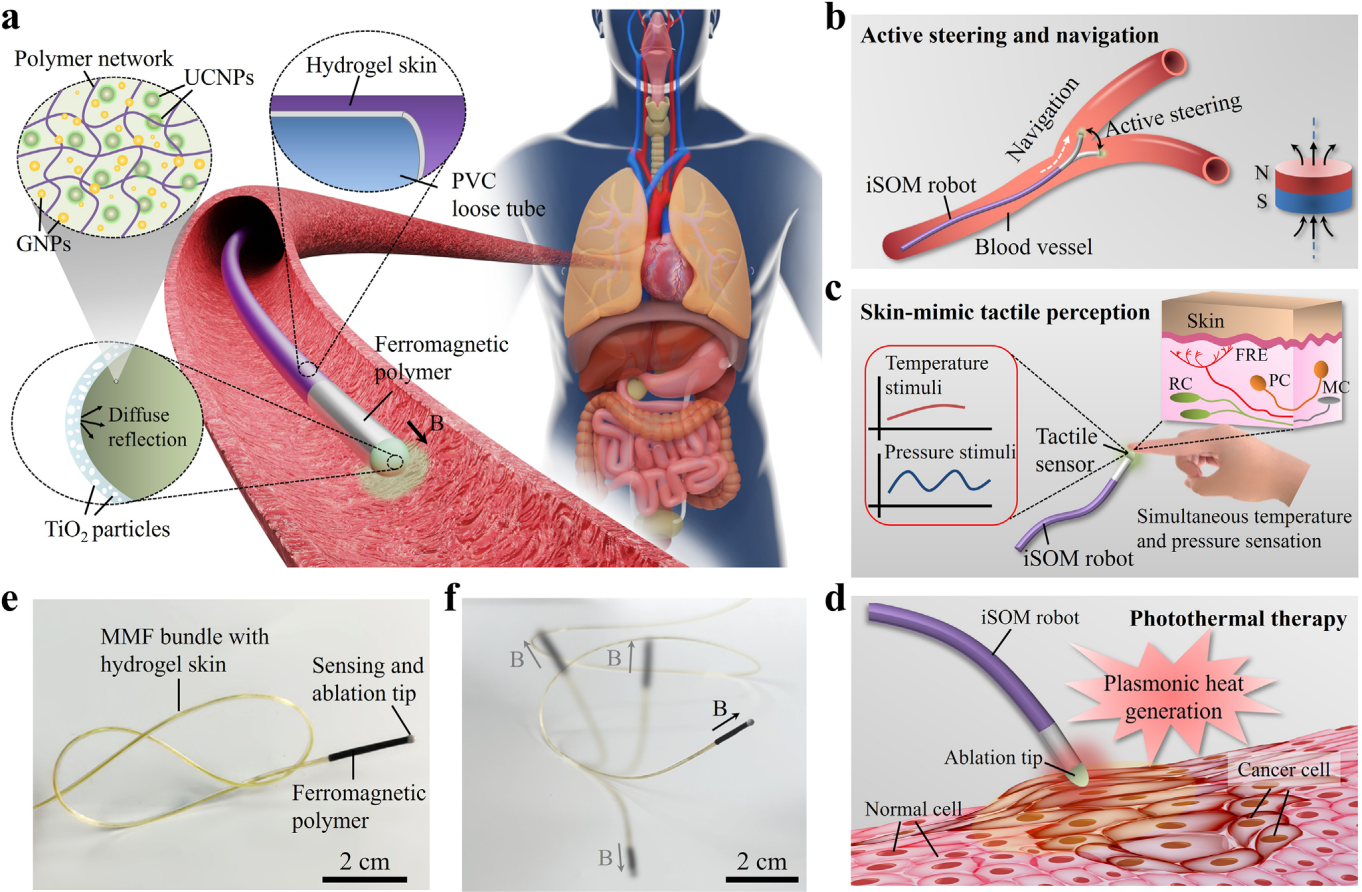
Design, structure, and functionality of the iSOM robot for minimally invasive interventional therapy. (a) Schematic illustration of the iSOM robot navigating confined anatomical spaces for targeted photothermal ablation. The robot comprises a bundle of multimodal optical fibers (MMFs) enclosed in a soft PVC sheath for light delivery and collection. The ferromagnetic polymer coated at the distal end of the robot enables magnetic steering, while the hydrogel skin on the outer surface serves as a self‐lubricating layer to reduce friction. The hemispherical tip, made from composites of UCNPs, GNPs, and PDMS elastomers, allows both multimodal tactile sensing and photothermal ablation, with a reflective TiO_2_/PDMS coating to prevent light leakage by diffuse reflection. (b–d) Functional versatility of the iSOM robot, (b) integrating magnetic steering, (c) skin‐mimic tactile perception, and (d) photothermal ablation at the millimeter scale. (e) Mechanical flexibility of the robot. (f) Omnidirectional steering of the robot upon magnetic actuation.

The iSOM robot, integrating multimodal tactile sensing, photothermal ablation, and magnetic actuation, can actively navigate through narrow and constrained natural cavities (e.g., cardiovascular system, bronchi, gastrointestinal tract, and bladder) within the human body to perform targeted TAT in a minimally invasive manner (Figure 1). The main body of the robot comprises a soft polyvinyl chloride (PVC) sheath that encases a bundle of multimodal optical fibers (MMFs), enabling both the excitation and collection of sensing fluorescence signals, as well as the delivery of optical therapeutic energy. The distal segment of the robot is coated with a ferromagnetic polymer comprising PDMS and uniformly dispersed NdFeB microparticles, fabricated through mold injection and thermal curing (see Figure S1a and Methods for details). To achieve the desired magnetic responsiveness, a strong magnetic field (∼3 T) was applied to align and magnetically saturate the NdFeB particles along the axial direction. The process of magnetization aligns the NdFeB particles to form stable magnetic dipoles, enabling controlled steering and navigation of the robot under an applied magnetic field (Figure 1b). To mitigate potential tissue damage due to friction during navigation, a hydrogel skin of high‐water content is applied to the robot's surface through an interfacial interpenetration strategy (Figure S1b) [27]. The hydrogel skin serves as a self‐lubricating layer that effectively reduces the surface friction while enhancing the robot's biocompatibility.

The incorporation of skin‐like tactile perception in surgical robots is crucial to achieving precise tissue manipulation, adaptive interaction, and minimized risk of injury to surrounding tissues. Human skin relies on specialized receptors to detect mechanical and thermal stimuli: slow‐adapting mechanoreceptors like Ruffini corpuscles (RC) for static touch, fast‐adapting mechanoreceptors such as Meissner's corpuscles (MC) and Pacinian corpuscles (PC) for vibrations, and free nerve endings (FRE) for temperature [28]. Mimicking these functionalities, the tip of the MMFs was designed with a hemispherical optical structure made from composites of UCNPs, GNPs, and PDMS elastomers, providing skin‐like softness, deformability, and multimodal tactile sensing. To prevent light leakage and enhance reflection, the hemispherical surface is coated with a high‐reflective PDMS film embedded with micrometric TiO_2_ particles. The soft tactile sensor enables simultaneous detection and discrimination of pressure and temperature through a plasmon–upconversion coupling mechanism, offering real‐time tactile feedback during magnetic‐actuated navigation (Figure 1c). Upon reaching the lesion site, light absorption by GNPs triggers localized surface plasmon excitation, driving efficient photothermal conversion for TAT of diseased tissues such as cancer (Figure 1d). Precise control of temperature and pressure is essential to ensure the safety and efficacy of thermal ablation. Excessive heat may cause unintended damage to adjacent healthy tissue, whereas insufficient temperatures risk incomplete ablation of the lesion  [29]. Pressure monitoring ensures proper contact between the ablation probe and the tissue. Inadequate contact force can impair energy delivery, while excessive or prolonged pressure may induce local tissue edema, mechanical injury, or compression‐related damage [30]. Through integrated multimodal sensing, the robot enables real‐time monitoring of both temperature and pressure during TAT, ensuring controlled, targeted therapy. The fabricated iSOM robot, with a diameter of ∼1.5 mm and a length of 25 cm, is soft, flexible, and capable of omnidirectional steering upon magnetic actuation (Figure 1e,f).

### 2.2 Highly‐Sensitive LSPR‐Based Pressure Sensing

**FIGURE 2:**
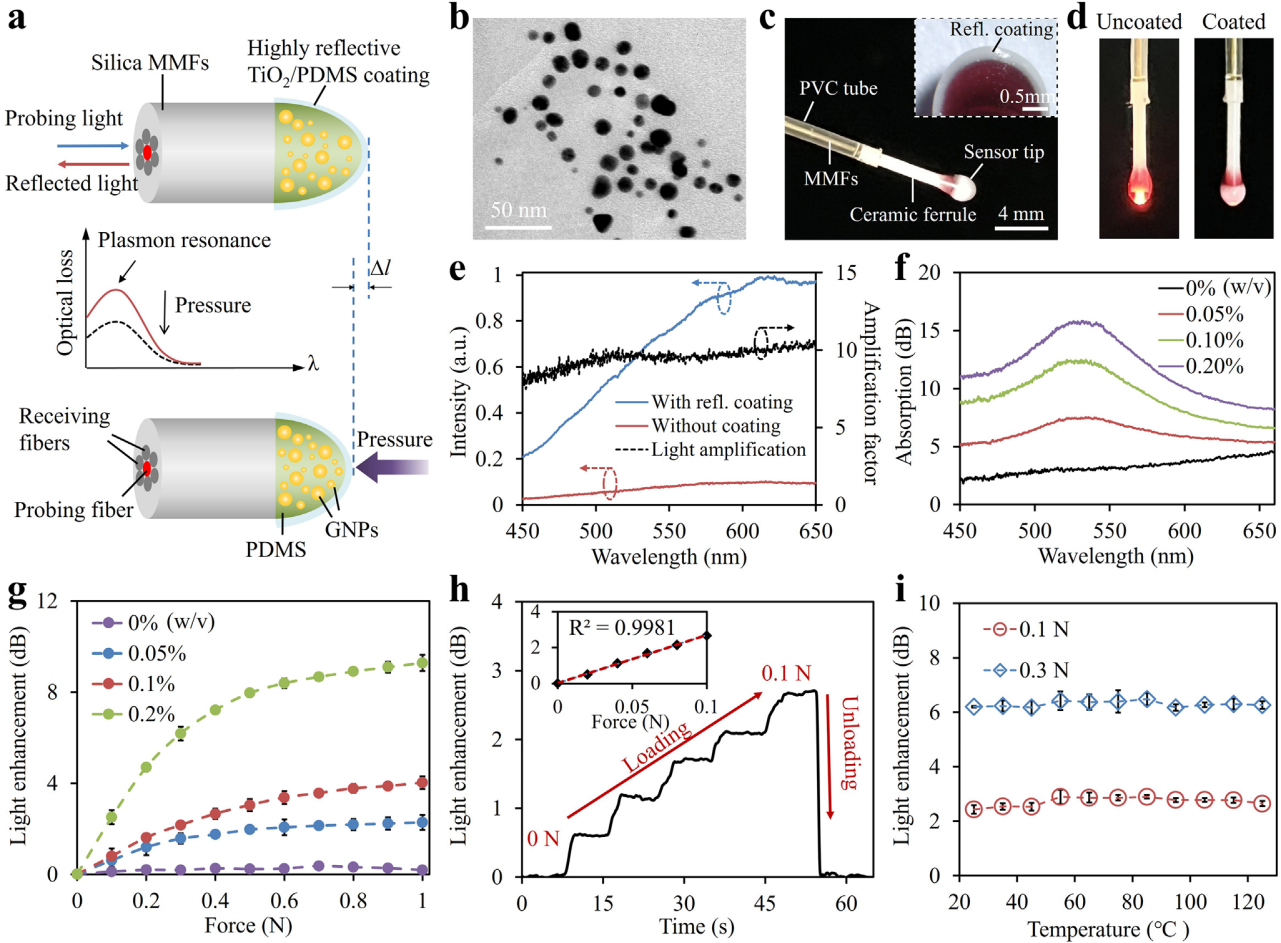
LSPR‐based pressure sensing of the iSOM robot. (a) Mechanism of pressure sensing based on LSPR effect. (b) TEM image of GNPs/PDMS composite. (c) Photograph of the sensor tip. The inset shows the cross‐section image of the tip under optical microscopy. (d) Photographs of the sensor tip before and after reflective coating under white light illumination. (e) Spectra of the reflected light with/without coating (left axis), along with the amplification factor provided by the reflective film (right axis). (f) Absorption spectra of the sensor doped with different GNPs concentrations. (g) Enhancement in reflected light versus applied pressure at different GNP doping levels (n = 3). (h) Temporal readout of the sensor under incremental pressure (0–0.1 N). The inset shows a linear fit of the sensor response. (i) Influence of temperature on the pressure response (n = 3).

The hemispherical structure at the tip of the MMF bundle was sensitive to pressure attributed to the intense LSPR effects of GNPs dispersed in the PDMS matrix (Figure 2a). The GNPs were synthesized in situ within the PDMS via reduction of gold salt (HAuCl_4_), with the Si‐H groups in the PDMS serving as the reducing agent (Figure S2) [31]. Transmission electron microscopy (TEM) revealed uniformly dispersed GNPs (∼8.4 nm) embedded in the PDMS matrix (Figure 2b). The fiber bundle consists of a central excitation fiber surrounded by six symmetrically arranged collection fibers. The probing light at the resonance wavelength of the GNPs transmitted through the dispersed particles and was absorbed through LSPR, resulting in significant attenuation detected by the receiving fibers. Under applied pressure, radial compression of the soft tip shortened the interaction length of light with the GNPs, resulting in reduced attenuation and enabling pressure quantification from changes in reflected intensity. To enhance reflectivity, the surface of the hemispherical tip was dip‐coated with an elastic, highly reflective TiO_2_/PDMS film (thickness, ∼250 µm) (Figure 2c; Figure S3). This reflective layer was covalently bonded to the tip surface and retained elasticity and deformability comparable to pure PDMS, ensuring stable adhesion even under vigorous deformations (Figure S4). The concentration of TiO_2_ was optimized at 4.5% w/v, providing a high reflectance of over 70% at wavelengths beyond 500 nm (Figure S5). This high reflection is attributed to the refraction and scattering of light resulting from the substantial differences in the refractive indices (*n*) of PDMS (*n* = 1.4) and TiO_2_ (*n* = 2.64) [32]. Figure 2d shows photographs of the sensor tip before and after coating with the reflective film when white light was launched. It can be observed that the coating effectively confines the injected light within the tip, thereby providing strong resistance against environmental interference (Figure S6). Spectral analysis of the reflected light further confirmed an approximately 10‐fold enhancement in reflected signal intensity across the visible range following coating (Figure 2e).

The absorption spectra of the sensor were measured as shown in Figure 2f. The GNP‐doped sensor showed a distinct peak at ∼532 nm, corresponding to the surface plasmon resonance of embedded GNPs. Increasing the GNP concentration intensified resonance absorption due to the amplified LSPR effect. To assess pressure responsiveness, a 1‐mW probing laser at 532 nm was launched into the sensor, and the reflected light was detected by a photodetector. As shown in Figure 2g, the reflected intensity increased with applied pressure, and higher GNP concentrations resulted in greater sensitivity due to enhanced plasmonic absorption. However, when the concentration increases beyond ∼0.2 wt%, excessive loading disrupts polymer crosslinking and compromises mechanical stability, while also causing markedly increased optical attenuation in the visible regime, reducing the detectable optical signal. Cyclic loading tests confirmed stable and repeatable responses with minimal hysteresis or drift, indicating reliable mechanical performance (Figure S7a). Due to its low elastic modulus, even small pressure applied to the sensor tip can induce significant deformation, allowing it to detect subtle pressure changes. Figure 2h shows the temporal readout of the sensor in response to stepwise increased pressure within a small range of 0–0.1 N. The concentration of GNPs was set to 0.2% w/v for its high sensitivity. The sensor showed an immediate and linear response to each pressure increment, with full signal recovery upon unloading. A noise‐limited detectable pressure as low as 4.5 mN was determined based on the standard deviation of the noise signal (Figure S7b). Response speed is another critical factor for dynamic measurements. The sensor's response and recovery times, defined as the duration required to reach 90% of the final value from the initial state, were measured to be 5 and 3 ms, respectively, surpassing the response speed of human skin (30–50 ms) (Figure S7c) [33]. Long‐term durability was validated by consistent performance over 10 000 loading‐unloading cycles (Figure S8). Moreover, the influence of temperature on the pressure sensing performance was investigated (Figure 2i). The sensor maintained a stable pressure readout across different temperatures, indicating thermal independence in its pressure response.

### 2.3 Simultaneous Detection of Temperature and Pressure

**FIGURE 3:**
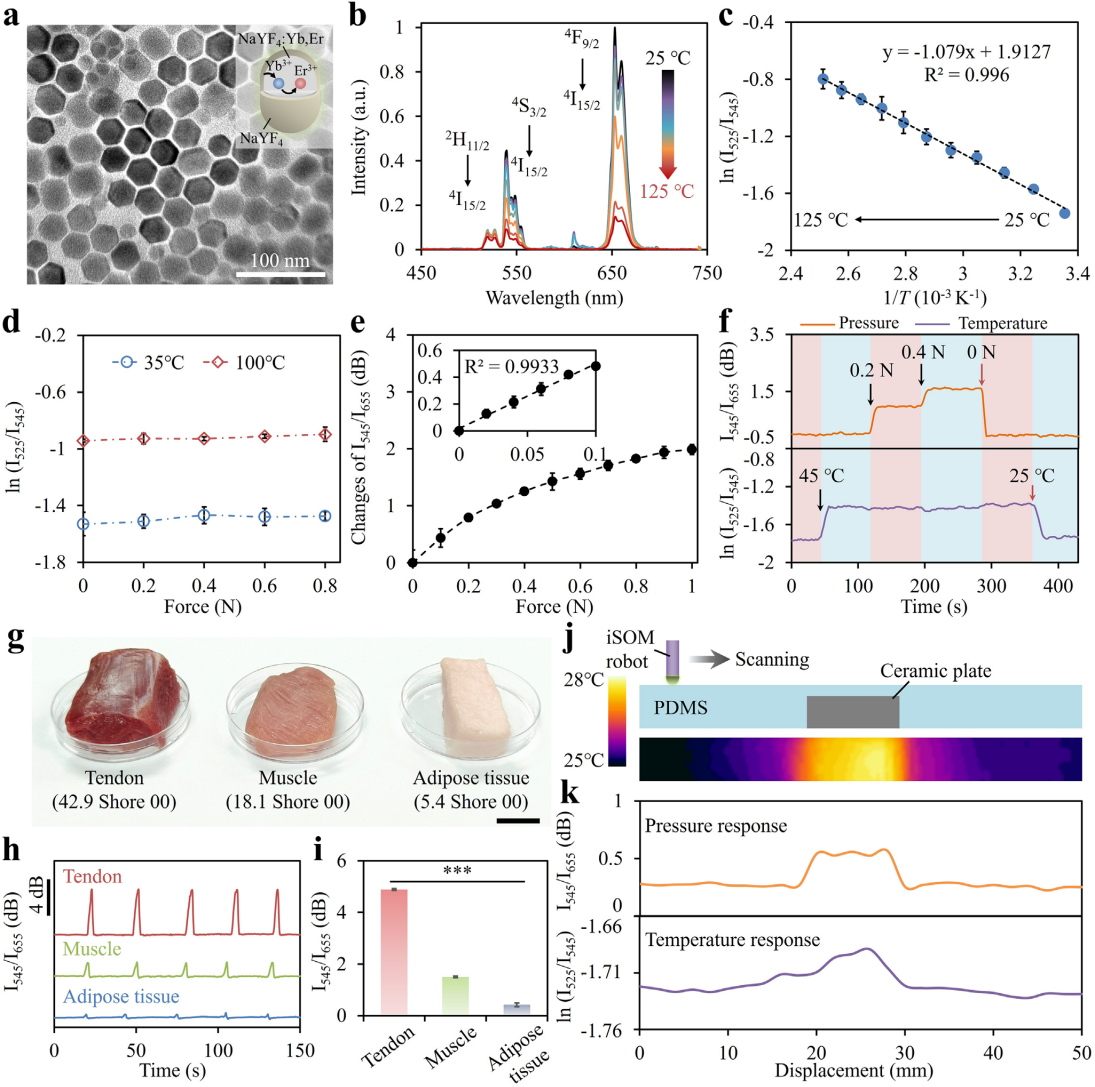
Simultaneous detection of temperature and pressure. (a) TEM image of the UCNPs. (b) Emission spectra of the sensor under different temperatures. (c) Linear plot of ln (*I*_525_/*I*_545_) versus 1/*T*. (d) Influence of pressure on the temperature response (n = 3). (e) Decibel scale changes of I_545_/I_655_ in relation to pressure (n = 3). The inset shows a linear fit of the sensor response over a small pressure range of 0–0.1 N. (f) Real‐time output of the sensor under combined stimuli of pressure and temperature. (g–i) Hardness perception and tissue discrimination through palpation. (g) Photographs of porcine tissues with varying hardness. Scale bar, 2 cm. (h) Real‐time pressure response during tapping palpation. (i) Peak pressure recorded for different tissues (n = 3). ^***^ *p* < 0.001 (student's t test). (j) Detection of hidden hardness and temperature abnormalities in a soft PDMS phantom. Top: schematic of the setup and phantom structure. Bottom: IR image of the phantom surface, showing a maximum temperature difference of ∼2.5°C. (k) Pressure and temperature signals during the robot scanning.

To achieve simultaneous detection of temperature, thermal sensitive Ln‐UCNPs were synthesized and incorporated into the sensor design. The UCNPs consists of a luminescent NaYF_4_:Yb,Er core encapsulated by an inert NaYF_4_ shell, which served to passivate surface defects and suppress environmental quenching [34]. TEM analysis confirmed a uniform hexagonal morphology with an average particle size of ∼28.3 nm (Figure 3a; Figure S9). The emission spectra of the sensor were analyzed at different temperatures using the optical setup depicted in Figure S10. A 980 nm NIR laser with a power of 10 mW was directed into the sensor, and the reflected emissions were collected for spectral analysis with a spectrometer. Upon NIR excitation, the incorporated UCNPs produced characteristic emissions at 525, 545, and 655 nm, corresponding to the Er^3+^ transitions of ^2^H_11/2_→^4^I_15/2_, ^4^S_3/2_→^4^I_15/2_, and ^4^F_9/2_→^4^I_15/2_ levels, respectively (Figure 3b). The emission intensities exhibited strong temperature dependence due to thermal coupling between the closely spaced ^2^H_11/2_ and ^4^S_3/2_ levels, which follow a Boltzmann distribution at thermal equilibrium [35]:

(1) $$I_{525}/I_{545}=A\exp\left(-\Delta E/kT\right)$$

where *I*_525_ and *I*_545_ represent the emission intensities of the ^2^H_11/2_→^4^I_15/2_ and ^4^S_3/2_→^4^I_15/2_ transitions, respectively; *A*is a constant; Δ*E* denotes the energy gap between the ^2^H_11/2_ and ^4^S_3/2_ levels; *k* is the Boltzman's constant, and *T* is the absolute temperature. As anticipated from Equation (1), a linear relationship betweenln (*I*_525_/*I*_545_) and the inverse temperature (1/*T*) was observed over the temperature range of 25°C–125°C (Figure 3c). The detection limit was determined to be 0.4°C, calculated from the standard deviation of the signal drift around 25°C (Figure S11a). To assess the repeatability of the temperature response, multiple thermal cycles involving sequential heating and cooling were conducted (Figure S11b). The sensor demonstrated consistent and reproducible readings throughout the temperature cycles, highlighting its stability and reliability. Response dynamics were further characterized under quasi‐transient temperature fluctuations, yielding response and recovery times of 6 s and 8 s, respectively (Figure S11c). Furthermore, the temperature response was insensitive to pressure due to the ratiometric dual‐wavelength detection, which inherently provides self‐calibration and robustness against interferences from other stimuli (Figure 3d).

With the multiband emissions generated through upconversion processes, simultaneous and decoupled sensing of temperature and pressure can be achieved under single NIR excitation. The emission at 545 nm, close to the LSPR peak of GNPs was chosen as the probing light, while the 655 nm emission, situated outside the plasmon resonance, was used as the reference. Figure 3e shows the decibel scale changes of I_545_/I_655_ in relation to pressure, revealing a trend similar to that obtained with a 532 nm laser (Figure 2g). To further demonstrate its capability for stimulus discrimination, the sensor was tested under various pressure and temperature conditions. As shown in Figure 3f, the sensor was able to detect real‐time changes in pressure and temperature independently, with negligible crosstalk, highlighting the sensor's robust and selective response. To our knowledge, this is among the few studies demonstrating simultaneous pressure and temperature detection within a single sensor architecture that combines skin‐like compliance and a compact form factor. Beyond enhancing navigation, the multimodal tactile sensing can also facilitate locating and identifying abnormal tissues during diagnosis and surgery. For example, tumors typically exhibit increased hardness and elevated temperature compared to normal tissues, making them distinguishable through tissue palpation [36, 37]. To evaluate the sensor's capability for hardness perception, it was employed to perform tapping‐based palpation on porcine tissues with different hardness (i.e., tendon, muscle, and adipose tissue) (Figure 3g). Upon tapping, the sensor tip induces both vertical and lateral tissue deformation, dissipating compressive energy. Tissues with higher hardness resist deformation and dissipate less energy, resulting elevated contact forces (Figure 3h). This mechanism enables the discrimination of tissues based on the peak pressure recorded during tapping palpation (Figure 3i). Additionally, a soft PDMS phantom embedded with a central ceramic heating plate was fabricated to mimic tissue regions with hidden hardness and temperature abnormalities. The heated plate produced a local temperature elevation of ∼2.5°C relative to the background (Figure 3j). The sensor tip was indented into the phantom to a fixed depth of 700 µm and scanned in 2 mm increments. As shown in Figure 3k, both pressure and temperature signals increased when the sensor traversed the embedded plate. The relatively larger area of the temperature anomaly, compared to that of the pressure response, was attributed to heat diffusion from the center, which slightly elevated the surrounding temperature. These results demonstrated the robot's potential to locate and identify hidden tissue abnormalities by simultaneously measuring thermal and mechanical characteristics, thereby facilitating precise diagnosis and treatment in minimally invasive surgery.

### 2.4 Real‐Time Tracked Photothermal Ablation Therapy

**FIGURE 4:**
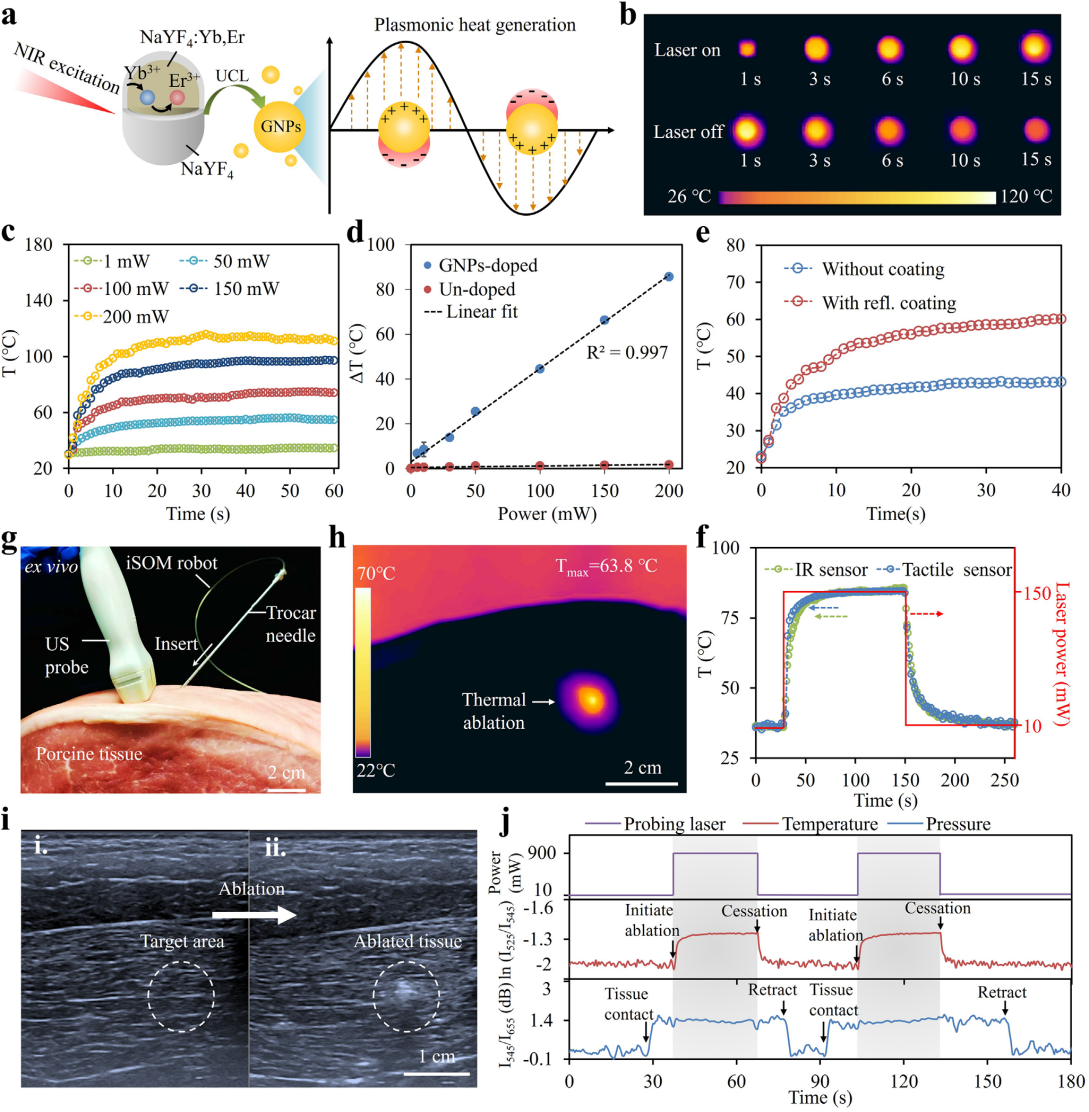
Real‐time tracked photothermal ablation therapy. (a) Mechanism of photothermal conversion based on LSPR upon NIR excitation. (b) IR thermograms of the ablation tip over time with the laser on and off. (c) Time‐elapse temperature increase of the ablation tip under different laser power levels. (d) Changes in temperature as a function of laser power with and without GNPs (n = 3). (e) Photothermal performances of the ablation tip with and without reflective coating. (f) Real‐time temperature changes captured by our sensor and an IR camera during the laser switching cycle between 10 and 150 mW. (g) Percutaneous insertion of the iSOM robot into porcine tissue through a trocar needle. Ultrasound (US) probe was employed to monitor tissue changes during ablation. (h) IR thermograms of the porcine tissue after laser heating at a power of 0.9 W for 1 min. (i) Ultrasound images of porcine tissue before and after ablation. (j) Real‐time pressure and temperature signals were acquired during the puncture and ablation processes, with two repeated trials performed to ensure measurement stability and reproducibility.

Upon NIR excitation, the UCL emissions, falling within the resonance range of gold nanoparticles (GNPs), are absorbed and converted into heat via the excitation of localized surface plasmons (Figure 4a). This process enables targeted photothermal ablation therapy by inducing cellular apoptosis and necrosis in diseased tissues, such as tumors, hemangiomas, and calcifications. The photothermal performances of the iSOM robot were investigated using a commercial IR camera, powered by a 980 nm laser. Figure 4b shows thermal images of the ablation tip over time with the laser on and off. Notably, the tip surface was observed to be uniformly heated, reaching temperatures above 70°C in 6 s at a laser power of 200 mW. After turning off the laser, the temperature rapidly decreased to below 45°C in 15 s. Additionally, increasing the laser power elevated both the temperature and heating rate (Figure 4c). The ablation temperature could be precisely controlled via the laser power, showing a linear correlation between power input and temperature rise (Figure 4d). The GNPs‐doped tip exhibited a significant temperature increase of approximately 86°C at a pumping power of 200 mW, whereas the undoped tip showed only a ∼1.7°C rise under the same conditions. Furthermore, the high‐reflective coating on the tip surface was shown to play a key role in enhancing the photothermal conversion efficiency (Figure 4e). This enhancement was attributed to the coating's ability to efficiently trap the laser beam that prolonged the interaction length between the excitation light and the GNPs. The photothermal process was continuously monitored in real time by the integrated sensor, enabling closed‐loop temperature control. Figure 4f shows temperature profiles recorded by both the sensor and an IR camera during a laser switching cycle between 10 and 150 mW. At the low setting of 10 mW, sufficient fluorescence intensity was generated for temperature sensing without causing noticeable heating. The sensor's readout closely matched that of the IR camera, validating the sensor's capability to precisely track dynamic temperature changes at the ablation tip.

To further evaluate its feasibility for photothermal ablation therapy, the iSOM robot was percutaneously inserted into porcine tissue at a depth of ∼2.5 cm through a trocar needle (inner diameter: 1.7 mm) (Figure 4g). Ultrasound imaging was employed to monitor tissue changes during ablation. Following laser heating at 0.9 W for 1 min, the target tissue temperature reached ∼63.8°C (Figure 4h; Movie S1). As irreversible thermal damage typically occurs above 50°C in diseased tissues such as cancerous lesions, this result demonstrates the robot's efficacy for photothermal ablation therapy. The ablation process alters the acoustic impedance of the target tissue due to thermal denaturation and coagulation, leading to changes in echo intensity [38]. Figure 4i shows ultrasound images of porcine tissue before and after ablation, where the target area appears hyperechoic and clearly distinguishable from the surrounding tissue, confirming successful ablation confirming successful ablation. Additionally, key parameters, including temperature and pressure, can be simultaneously monitored during ablation. Figure 4j demonstrates distinct, repeatable signal changes aligned with each procedural phase. Pressure monitoring ensured stable tissue contact, while temperature feedback enabled precise thermal regulation to prevent collateral damage. To further quantitatively assess the effective treatment range of photothermal ablation, the ablation depth and lateral extent were systematically characterized in ex vivo tissue samples (Figure S12). Both the penetration depth and cross‐sectional area of the ablated region increased consistently with increasing laser power, indicating that the ablation geometry can be predictably modulated by adjusting the delivered optical energy.

### 2.5 Low‐Friction Magnetic Guidance of the Robot

**FIGURE 5:**
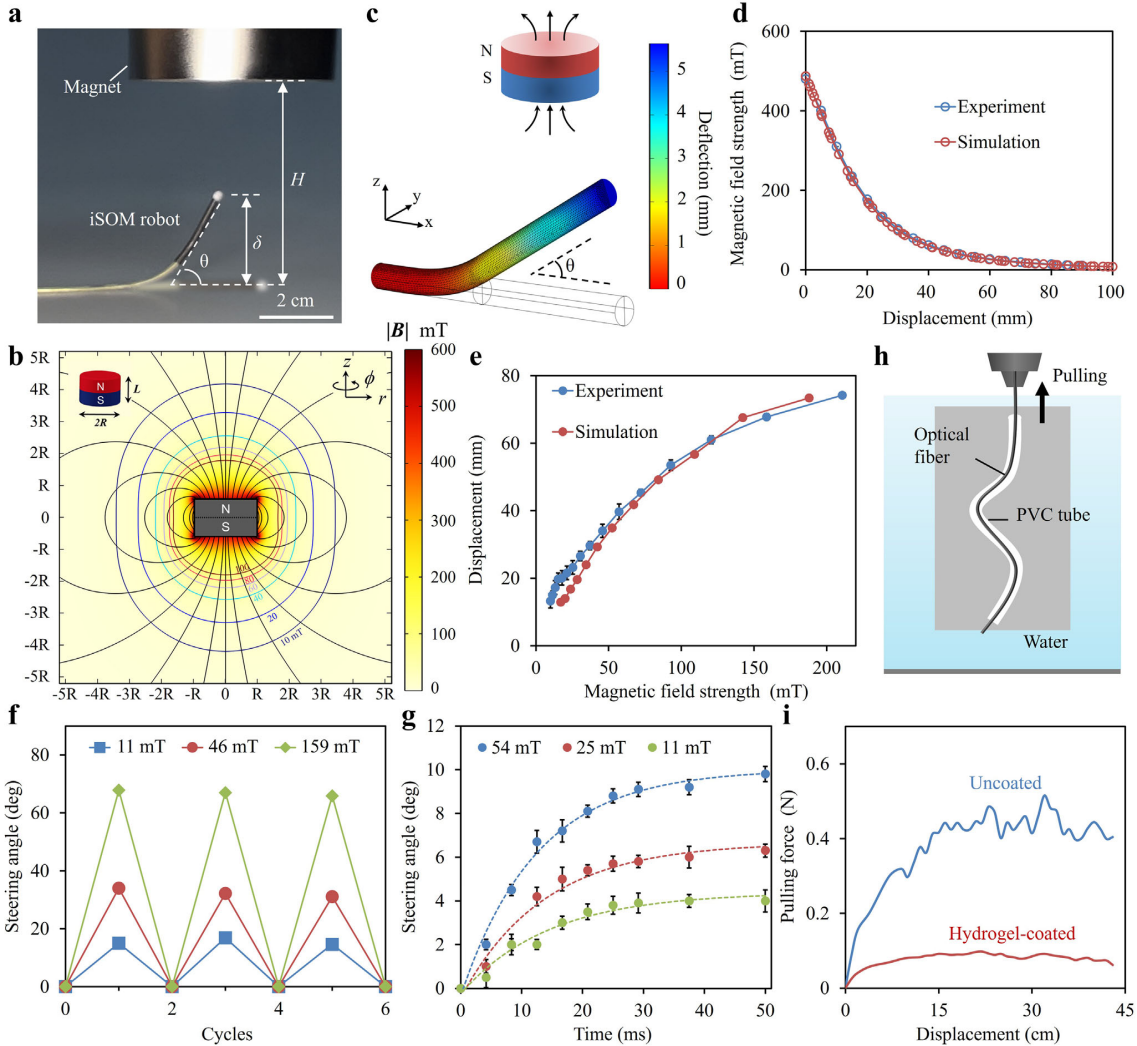
Low‐friction magnetic guidance of the robot. (a) Experimental setup for evaluating the magnetic responsiveness of the iSOM robot. (b) Axisymmetric magnetic field distribution simulated for an N42 cylindrical magnet (diameter 2R = 50 mm, thickness L = 30 mm, remanence Br = 1.32 T). (c) FEM simulation of the robot deflection under external magnetic actuation. (d) Comparison of magnetic field strength at the robot tip from experiments and simulations at different working distances. (e) Relationship between the steering angle and magnetic field strength obtained from experiments and simulations (n = 3). (f) Multiple cycles of actuation tests under different magnetic field strength, indicating repeatability and stability of magnetic steering. (g) Time‐resolved tip displacement under varying magnetic field strengths (n = 3). (h) Schematic of friction test, where the robot was pulled through a soft, tortuous tube with a narrow inner diameter of 2 mm. The robot's tip was clamped onto a force gauge and pulled at a constant speed. (i) Pulling force versus displacement for hydrogel‐coated and uncoated robots.

To enable remote‐controlled guidance via magnetic actuation, the iSOM robot was coated with PDMS elastomer embedded with axially magnetized NdFeB particles. Magnetic responsiveness was characterized using a cylindrical N42 permanent magnet (5 cm diameter, 3 cm thickness) that provided a nonuniform magnetic field for actuation (Figure 5a). The magnet working distance *H* is defined as the distance from the robot tip to the bottom of the magnet and δ indicates the tip deflection. By adjusting 𝐻, the magnetic field surrounding the robot is altered, driving it to deform and steer to a specific angle (θ). With the application of such a nonuniform magnetic field, the embedded NdFeB particles experience magnetic torque ***τ*** and magnetic force ***f***, described as follows:

(2) τ=M×B

(3) $$f=\left(M•\nabla \right)B$$

where **M** denotes the magnetization of the robot tip along its axial direction, **B** is the external magnetic field, and ∇**B** represents the spatial gradient. The resulting magnetic torque and force induce a magnetic Cauchy stress that drives deformation of the robot, which can be expressed as [39]:

(4) σ=−B⊗FM

where **F** is the deformation gradient, and the operation⊗denotes the dyadic product, which combines two vectors to form a second‐order tensor. Based on the magnetic Cauchy stress implemented in the COMSOL Multiphysics platform, we simulated the deformation of the robot using a 3D finite element model (FEM) that approximates the presented design (Figure 5b,c and see Supporting Information for more details). The magnetic field applied in the simulations matched the experimental conditions and was validated against measured field distributions (Figure 5d). The relationship between steering angle and magnetic field strength was determined both experimentally and computationally, showing good agreement (Figure 5e). The measured steering angle increased monotonically with the applied field, following the trend predicted by the FEM. Repeated actuation tests under different magnetic field strengths demonstrated a high steering precision of ∼1.5° (Figure 5f). The response speed of the robot upon magnetic actuation was further assessed with a high‐speed camera, indicating a rapid response time as low as 25 ms (Figure 5g). These results confirmed the fast, precise, and reliable steering capabilities of the robot through the manipulation of magnetic field.

To reduce frictional resistance during navigation, particularly in confined or tortuous environments, a thin hydrophilic polyacrylamide (PAM) hydrogel layer was covalently grafted onto the robot's PVC sheath. The PVC surface was first swollen by immersion in an ethanol‐based solution containing hydrophobic photoinitiators (benzophenone), which facilitated the diffusion of initiators into the polymer matrix (Figure S1b). The treated sheath was then immersed in a PAM precursor solution containing hydrophilic photoinitiators (Irgacure 2959). Under ultraviolet (UV) irradiation, the hydrophilic photoinitiators initiated the photocrosslinking of PAM monomers, while the hydrophobic initiators enabled the PAM networks to covalently crosslink with the PVC chains, resulting in a firmly attached hydrogel layer. Microscopy images confirmed the presence of the hydrogel skin on the robot surface (Figure S13a). The coating, stained with Rhodamine B, exhibited a uniform thickness of 30–50 µm. The lubricity of the hydrogel markedly reduced the surface friction of the robot by over 70%, as measured by a friction tester (Figure S13b,c). Frictional performance was further assessed by pulling the robot through a soft, tortuous tube with a 2 mm inner diameter, mimicking navigation in confined anatomical environments (Figure 5h). The robot tip was clamped onto a force gauge and pulled at a speed of ∼23 mm/min. The results show that the pulling force required by the hydrogel‐coated robot was reduced by four times, demonstrating the coating's effectiveness in minimizing friction and enabling smoother navigation (Figure 5i). Besides, the hydrogel skin endowed the robot with good biocompatibility, as evidenced by in vitro cell viability assays (Figure S14) and in vivo local biosafety evaluation in mice, where no exacerbated inflammatory response or abnormal pathological changes were observed compared with control tissue (Figure S15).

### 2.6 Active Navigation and Cardiac Ablation in Cardiovascular Phantoms

**FIGURE 6:**
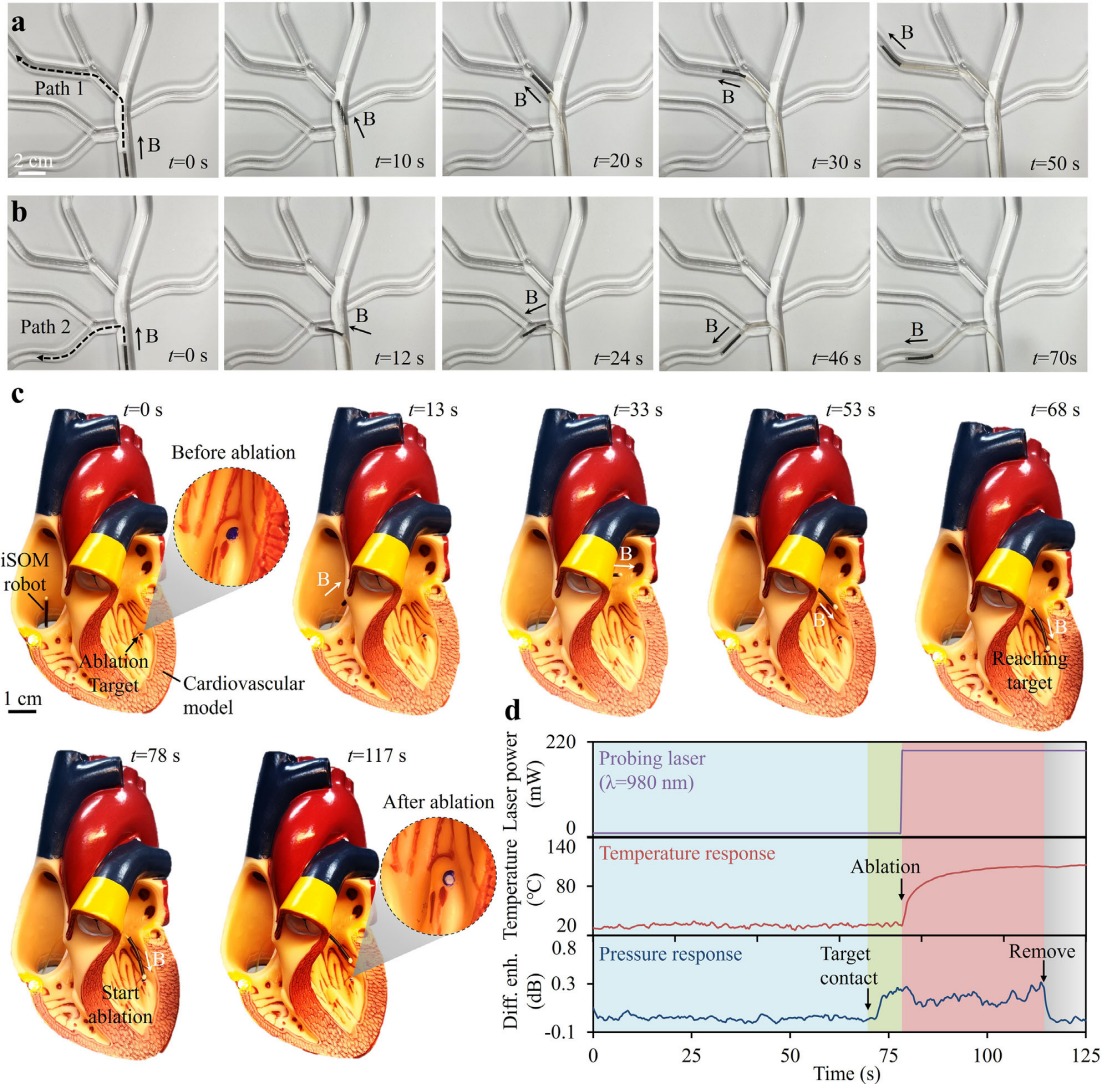
Active navigation and cardiac ablation therapy in cardiovascular phantoms. (a) Navigation through a tree‐structured vascular phantom, performing forward and selective motion. The magnetic steering was achieved by manually adjusting the working distance and orientation of the permanent magnet, while the entire body was advanced by pushing the rear part of the robot. (b) Steering the robot to execute large‐angle deflections and backward motion. (c) Demonstration of the robot navigating within a cardiovascular phantom model to perform targeted ablation in the left ventricle via vascular access. (d) Real‐time pressure and temperature readouts of the robot, along with the probing laser power.

Benefiting from the novel synergy of materials, design, and functionality, the iSOM robot holds great promise for navigating natural orifices, such as organ or vascular lumens, to perform minimally invasive focal ablation. As proof of concept, we demonstrated the robot's ability to traverse complex vasculature and conduct targeted cardiac ablation in cardiovascular phantoms. A branched vascular phantom with sharp turns (up to 90°) and inner diameters of 5–10 mm was fabricated via 3D printing using polycarbonate and filled with deionized water to mimic intravascular conditions. The primary task of the iSOM robot was to navigate along predefined vascular paths toward target locations. Magnetic actuation was provided by a cylindrical permanent magnet (50 mm diameter, 30 mm height) positioned at working distances of 20–80 mm. By aligning the magnet's central axis with the target orientation, the robot tip was steered accordingly, while the full body was propelled forward through proximal pushing. Figure 6a shows the process of steering the robot through the vascular phantom, performing two targeted turns to reach side branches. It successfully passed through branch angles of 47° (t = 10 s), 37.4° (t = 30 s), and 42.2° (t = 50 s), covering a total distance of ∼15 cm. In addition to forward and selective turning, the robot also achieved 90° deflections and backward motion (Figure 6b). Backward motion is inherently challenging but crucial, as the target is not always located ahead. These experiments validate the iSOM robot's capability for precise, flexible navigation through constrained anatomical pathways under magnetic control (see also Movie S2).

Furthermore, we demonstrated that the iSOM robot enables precise, remote‐controlled cardiac ablation under magnetic actuation. Cardiac ablation is a minimally invasive procedure that involves puncturing a peripheral blood vessel and using a catheter to deliver thermal therapy to targeted cardiac tissue [40]. Despite its widely applications in clinical practice, this procedure heavily relies on the surgeon's expertise, demands exceptionally high precision, and carries significant risks. Real‐time monitoring of contact pressure and temperature at the ablation tip is therefore essential to enhance therapeutic efficacy and reduce surgical complications. Moreover, conventional cardiac ablation is typically performed with X‐ray guidance, exposing medical personnel to cumulative radiation and necessitating prolonged use of heavy lead aprons. The proposed robot addresses these challenges by enabling remote operation and real‐time intraoperative monitoring. Figure 6c shows the robot navigating through a cardiovascular phantom to perform targeted ablation in the left ventricle via vascular access. The target region was coated with thermochromic ink that changed color from blue above 55°C, providing a clear visual indicator of the thermal impact on the phantom tissue for evaluating ablation effectiveness (Figure S16). The robot entered the right atrium via the inferior vena cava (t = 13 s), traversed the atrial septum into the left atrium (t = 33 s), and passed through the left atrioventricular valve to reach the target ablation site in the left ventricle (t = 68 s). It accurately localized the target and achieved effective ablation, as indicated by the distinct color change in the thermochromic tissue (see also Movie S3). During the navigation, the probing laser of the robot operated at low‐power mode (∼10 mW), maintaining the minimum energy required for tactile sensing. Upon contact with the target tissue, the laser then switched to high‐power mode (∼200 mW), rapidly releasing heat to initiate TAT (t = 78 s). The temperature and pressure responses of the iSOM robot throughout the entire process are illustrated in Figure 6d, highlighting its capability to simultaneously monitor real‐time contact pressure and temperature variations at the ablation tip.

### 2.7 Ex/In Vivo Interventional Validation of the iSOM Robot

**FIGURE 7:**
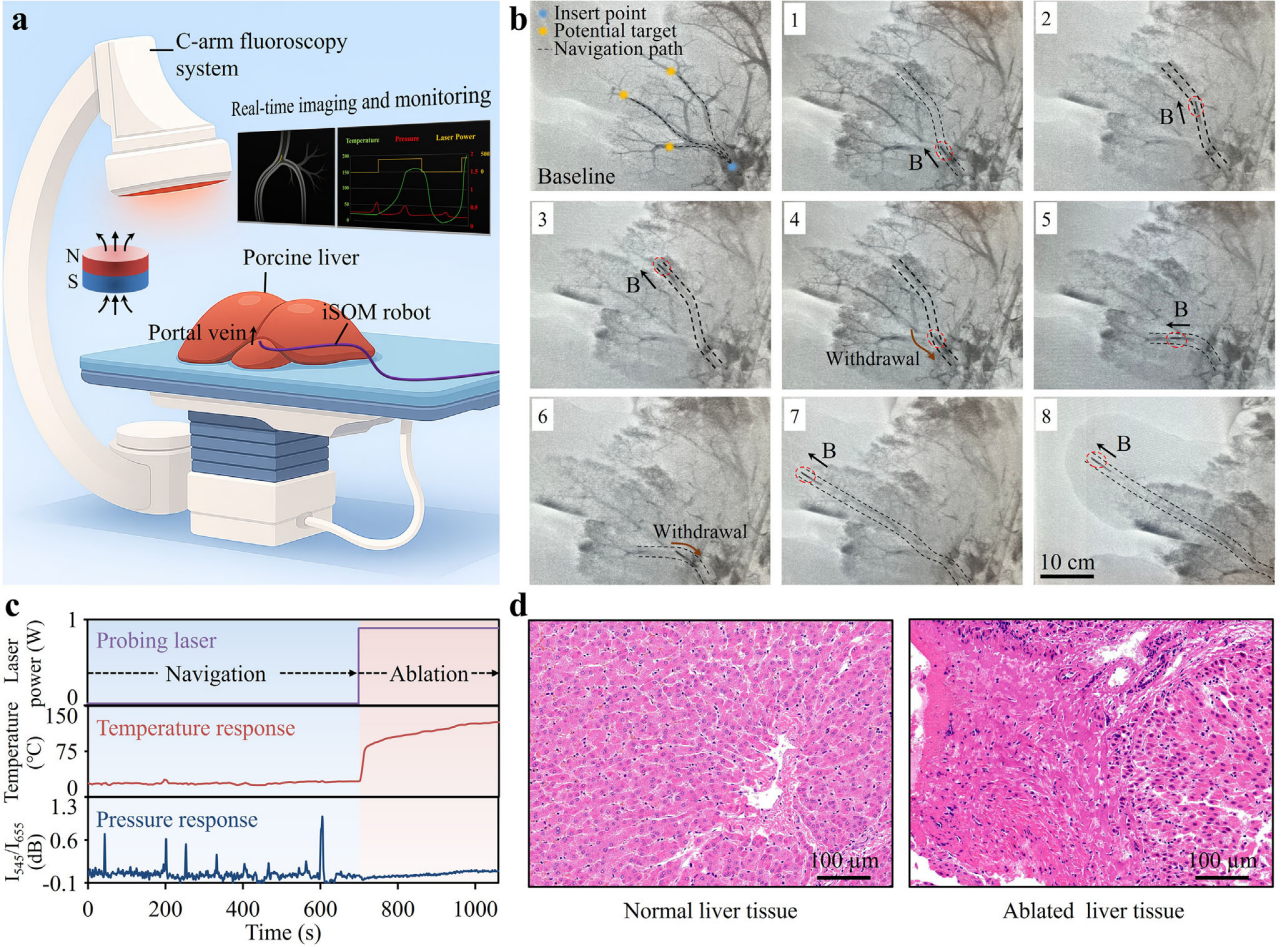
X‐ray guided interventions in the porcine hepatic portal venous system. (a) Illustration of the robot performing vascular interventions under X‐ray guidance with real‐time monitoring of temperature and pressure. The robot was introduced via the portal vein and steered by a permanent magnet. (b) Sequential X‐ray images showing the navigation of the robot in the porcine hepatic vasculature. The first image (baseline) was acquired immediately after contrast agent injection and used as a vascular reference. Subsequent frames capture the robot's progression toward intrahepatic targets under magnetic guidance. (c) Real‐time readouts of pressure, temperature, and probing laser power during the procedure. (d) Histological comparison between normal and ablated liver tissues.

Targeted ablation via the portal venous system offers unique clinical value in the management of hepatic malignancies. It holds particular relevance for treating hepatocellular carcinoma (HCC) adjacent to major portal branches, portal vein tumor thrombus (PVTT), and deep‐seated or residual metastatic lesions that are otherwise inaccessible by percutaneous or surgical approaches. To further assess the translational potential of the iSOM robot, ex vivo X‐ray‐guided vascular interventions were performed in porcine livers, with real‐time monitoring of local temperature and contact pressure for ensuring procedural safety and therapeutic precision (Figure 7a). Porcine livers were chosen for their anatomical similarity to human hepatic vasculature, particularly the portal venous system [41]. Prior to the procedure, iodinated contrast agents were perfused into the vasculature to enhance vessel visibility under X‐ray imaging. The initial post‐injection image was used as a vascular reference for navigation, as the contrast agent gradually diffused over time, reducing imaging clarity. The iSOM robot was introduced via the portal vein and magnetically guided through the branched vasculature, demonstrating the ability to turn, traverse bifurcations, and follow designated pathways under magnetic actuation (Figure 7b). It ultimately reached the distal end of the medial segment of the left hepatic lobe via the terminal branch of the left portal vein, where localized ablation was successfully performed at 0.9 W for 6 min. Figure 7c presents the robot's real‐time pressure and temperature readouts. Notably, distinct pressure spikes were observed during navigation, corresponding to transient contact or collision with the vessel wall. In contrast, the temperature readout remained stable during navigation but increased rapidly to a maximum of ∼120°C during ablation. These signals offer valuable intraoperative feedback for procedural monitoring and potential closed‐loop control. Subsequent histological analysis revealed a distinct morphological contrast between normal and ablated liver tissue (Figure 7d). Normal regions exhibited well‐preserved lobular architecture, with intact hepatocytes showing centrally located nuclei and uniform cytoplasmic staining. In contrast, ablated areas showed sharply defined zones of thermal injury, featuring patchy hepatocellular necrosis extending to vascular and connective tissue structures. Necrotic cells displayed nuclear pyknosis, karyorrhexis, and cytoplasmic fragmentation, forming amorphous eosinophilic debris. These observations underscore the therapeutic efficacy of the ablation and its ability to induce well‐localized tissue damage with minimal collateral effects. To further validate the iSOM robot under in vivo conditions, gastric interventions were performed in a rabbit under endoscopic guidance (Figure S17). A miniaturized endoscope was introduced through the oral cavity and advanced into the stomach, and the iSOM robot was delivered alongside the endoscope under continuous endoscopic visualization. After entering the gastric cavity, the robot was magnetically actuated to perform controlled forward and reverse deflections, demonstrating reliable intraluminal navigation with stable magnetic responsiveness and positional control despite intrinsic physiological movements such as respiration and gastric motility. Localized photothermal ablation was applied to the gastric wall while real‐time temperature and pressure signals were continuously recorded. These results confirm that the iSOM robot can operate robustly within a living luminal environment, demonstrating integrated navigation, sensing, and therapeutic capabilities.

## 3 Conclusions

In summary, we have presented a millimeter‐scale iSOM robot that integrates skin‐mimic multimodal tactile sensing, photothermal ablation, and magnetic actuation, along with key attributes of high flexibility, low‐surface friction, and biocompatibility. The robot's body consisted of a soft PVC sheath that encased bundled MMFs for light delivery and collection. The leading section of the robot was coated with ferromagnetic polymer to allow magnetic steering, while the front tip featured a hemispherical optical structure that enabled both tactile sensing and photothermal ablation. To mitigate friction during navigation, biocompatible hydrogel skin was applied to the robot surface to provide lubrication. By exploiting the plasmon‐upconversion coupling mechanism, the robot enabled real‐time, decoupled monitoring of temperature and pressure variations during tissue contact under single NIR laser excitation. Simultaneously, the excited LSPR was also harnessed to efficiently convert light energy into localized heat, facilitating targeted ablation therapy. The robot was validated through proof‐of‐concept demonstrations, including real‐time tracked percutaneous photothermal ablation in porcine tissue, targeted cardiac ablation in cardiovascular phantoms, and image‐guided therapeutic interventions in ex vivo and in vivo anatomical systems, highlighting its clinical potential in minimally invasive interventional therapies.

Compared to existing robotic technologies, the iSOM robot offers key advantages in miniaturization, multifunctional integration, and biomechanical compatibility. Its low‐profile design, combining photothermal ablation and magnetic actuation, enables remote‐controlled navigation in confined spaces for targeted therapy, while real‐time monitoring ensures precise and safe interventions. The soft, biocompatible structure and self‐lubricating surface enhance tissue compatibility, allowing it to conform to complex tissue surfaces and minimizing the risk of friction‐related injuries. For magnetic steering, a single permanent magnet was used to generate the required magnetic fields by manually adjusting its position and orientation. To further improve steering precision, more advanced magnetic field control systems, such as MRI or electromagnetic field generation systems, could be employed. MRI is particularly promising, as it facilitates both magnetic actuation and imaging guidance without ionizing radiation. Additionally, due to the small size and versatility of optical fiber, the functionality of the robot could be further enriched by integrating microscale fiber‐optic imaging technologies such as optical coherence tomography (OCT) or photoacoustic imaging (PAI), enabling real‐time tissue imaging during minimally invasive treatments. Overall, the iSOM robot is expected to offer an innovative, safer, and more efficient approach to minimally invasive therapies, with the potential to transform clinical practice across diverse medical applications.

## 4 Methods

### 4.1 Fabrication of the iSOM Robot

The main body of the iSOM robot featured a flexible PVC sheath (outer diameter: 1.2 mm) enclosing a bundle of MMFs, with a central fiber for laser excitation and six surrounding fibers for collecting emitted light. Each fiber had a core/cladding diameter of 100/110 µm and a numerical aperture (NA) of 0.22. The pristine body was first cleaned with a plasma cleaner (PDC‐001, Harrick Plasma) for about 2 min to remove surface contaminants and improve wettability. To enable magnetic responsiveness, the front section (∼2 cm) of the robot body was coated with a ferromagnetic polymer composite, achieved by inserting it into a polyethylene tube mold with an inner diameter of 1.5 mm. A precursor solution containing 30 vol% NdFeB powder (average particle size: 5 µm, Magnequench) was prepared by mixing it with PDMS (Sylgard 184, Dow Corning) at a base‐to‐curing agent ratio of 10:1. After vigorous stirring and degassing, the solution was injected into the mold via a syringe and magnetized along its axial direction under a strong magnetic field (∼3 T). The coated body was then cured at 80°C for 5 h and removed from the mold using water pressure. To form a hydrogel skin on the robot's outer surface, the robot was first treated with an ethanol solution containing 10 wt% benzophenone (Aladdin) for 1 min. After wiping off the excess solution, the robot was immersed in a hydrogel precursor consisting of 30 wt% acrylamide (Aladdin) and 1 wt% Irgacure 2959 (Aladdin) dissolved in deionized water. The solution was exposed to ultraviolet light (365 nm, 5 mW cm^−2^) for 60 min to polymerize the hydrogel onto the robot surface, then rinsed with deionized water to remove any residual reagents.

The robot tip was designed with a hemispherical optical structure made from composites of UCNPs, GNPs, and PDMS elastomers. The core–shell UCNPs were synthesized via a solvothermal method described in previous reports [42, 43], and the obtained nanoparticles were dispersed in cyclohexane for subsequent use. The GNPs were synthesized via in situ reduction by mixing gold salt (HAuCl_4_·3H_2_O, Sigma) with a pre‐prepared PDMS precursor (monomer/curing agent, 5:1). The precursor solution, co‐doped with 2 wt% UCNPs, was sonicated at 40 kHz in ice water for 30 min, followed by stirring for an additional hour. By applying ∼2 µL of the precursor solution to the robot tip, a hemispherical structure was formed vertically under gravity, followed by thermal curing at 120°C for about 30 min. The solidified tip was subsequently coated with a high‐reflectivity TiO_2_/PDMS film to minimize light leakage through dip‐coating. The coating solution was prepared by dispersing TiO_2_ particles (<5 µm, Sigma) in PDMS at a concentration of 4.5 wt%. The dipping thickness was controlled by the withdrawal speed and dipping time. Finally, the coating was cured at 120°C for 30 min.

### 4.2 Optical Setup for Tactile Sensing and Photothermal Ablation

A fiber‐coupled 980 nm laser was launched into the robot via the central MMF, and the reflected emissions were collected by six receiving fibers and routed to a spectrometer (CCS100, Thorlabs) for spectral analysis. A short‐pass filter with a cutoff wavelength of 850 nm was placed before the spectrometer to block the excitation laser. The laser power was adjusted based on different operational conditions. During robot navigation, the laser operated at a low power of ∼10 mW, providing the minimum energy required for tactile sensing. For photothermal ablation, the laser was switched to a high power of over 200 mW to generate localized high heat.

### 4.3 Instruments and Characterizations

TEM and SEM imaging were conducted using a JEM‐2100F TEM (JOL) and a Gemini SEM 560 (ZEISS). The absorption spectra of the sensor tip at various GNPs concentrations were measured using a white light source (Ocean Optics, HL‐2000) and a spectrometer. X‐ray diffraction (XRD) patterns of the UCNPs were investigated with a Bruker D8 diffractometer. The infrared spectra of TiO_2_/PDMS samples were analyzed by a Fourier transform infrared (FT‐IR) spectroscopy (Nicolet 6700). The mechanical properties of the samples were evaluated using a tensile tester (Handpi Instruments) with a 10 N load cell. The hardness of the porcine tissues was measured with a Shor 00 durometer. The temperature sensing performance of the robot was characterized by immersing the sensor tip into a heated water bath, with the temperature recorded using a digital thermometer with 0.1°C resolution. Pressure sensing capabilities were evaluated using a motorized loading system (Handpi Instruments), which was actuated through a signal generator (Keysight, DSOX1024G) and a broadband power amplifier (Aigtek, ATA‐1220D). Photothermal performance was investigated with a commercial IR camera (FLIR Pro) powered by a 980 nm laser. X‐ray imaging was performed with a C‐arm fluoroscopy system (INNOVA 3100, GE), which provided real‐time planar imaging for procedural guidance.

### 4.4 Friction Testing

The lubricating properties of the hydrogel were evaluated using a friction coefficient tester (PUYUN). PVC sheets (10 × 10 cm) were prepared with and without hydrogel coatings. The surface of each sample was wetted with deionized water. A 200 g mass was then drawn across the sample surface at a constant speed of 100 mm/min. The force required to slide the mass was measured for both the hydrogel‐coated and uncoated samples, and the friction coefficient was read from the tester.

### 4.5 Magnetic Actuation and Testing

A cylindrical permanent magnet (N42, diameter 5cm, 3 cm thickness) was used to actuate the iSOM robot. A Gauss meter (F.W.BELL 5180) was employed to measure the magnetic field of the cylindrical permanent magnet along the centerline at various working distance. The steering angle and defection displacement of the robot under different magnetic field strength were investigated with a high‐speed camera. Magnetic steering of the robot in cardiovascular phantoms and porcine hepatic vasculature was achieved by manually adjusting the magnet's position and orientation to alter both the direction and strength of the magnetic fields. The robot's forward motion was facilitated by advancing its rear section.

### 4.6 Animal Experiments

A New Zealand White rabbit (∼2.3 kg) was used for in vivo gastric interventional validation. Anesthesia was induced by intravenous injection of ketamine (35 mg/kg) and xylazine (3 mg/kg) via the marginal ear vein, and the animal was positioned in the lateral position on an operating platform. A medical‐grade miniaturized endoscope was inserted through the oral cavity and advanced along the esophagus into the stomach to provide real‐time visual guidance. The iSOM robot was introduced through the same oral pathway alongside the endoscope, with its position continuously monitored under endoscopic visualization until it reached the gastric cavity.

To evaluate in vivo local biosafety, BALB/c mice (15–17 g) were anesthetized by intraperitoneal injection of ketamine (80 mg/kg) and xylazine (10 mg/kg). After shaving and disinfecting the dorsal interscapular region, a small skin incision of approximately 5 mm was made, and a superficial muscle pocket was created by blunt dissection. The distal end of the iSOM robot was inserted into the superficial muscle layer and left in place for 30 min under short‐term interventional contact conditions. After removal of the robot, the skin incision was closed with sutures. One mouse was monitored for 1 week to assess survival status and wound healing. Another mouse was euthanized 24 h after the procedure for the collection of tissue samples and the assessment of acute local tissue responses by histology. A control mouse underwent the same dorsal incision and muscle pocket procedure, but without robot implantation. All animal procedures were reviewed and approved by the Institutional Animal Care and Use Committee (IACUC) of Beijing Tonghe Litai Biotechnology Co., Ltd.

### 4.7 Statistical Analysis

All quantitative data are presented as mean ± SD (unless otherwise noted), and the sample size (n) for each dataset is indicated in the corresponding figure legend. Group differences are analyzed using the student's t‐test (GraphPad Prism 7 San Diego, CA, USA). Results with *P* values of <0.05 are considered statistically significant.

## Author Contributions

J.G. and L.X. conceived the idea. J.G., J.Z., and X. J. designed the research. X.G. and J.W. performed the experiments. X.G., J.G., Q.S., and X.X. analyzed the data. B.F., Z.L., R.C., M.L., M.J., and J.M. contributed to experimental support. J.G. and X.G. wrote the manuscript with input from all authors.

## Conflicts of Interest

J.G., X.G., Z.L., and L.X., have pending patent applications on the presented design. The other authors declare no competing interests.

## Supporting information




**Supporting File**: advs73960‐sup‐0001‐SuppMat.docx

**Supporting File**: advs73960‐sup‐0002‐MovieS1.mp4

**Supporting File**: advs73960‐sup‐0003‐MovieS2.mp4

**Supporting File**: advs73960‐sup‐0004‐MovieS3.mp4

## Data Availability

All data needed to evaluate the conclusions in the paper are present in the paper and/or the Supplementary Materials.
