# Supplementary material for: Intelligent Soft Opto‐Magnetic Robot for Minimally Invasive Interventional Therapy
Source: Adv Sci (Weinh). 2026 Jan 21;13(18):e20344. doi: 10.1002/advs.202520344 (PMC13042443; doi:10.1002/advs.202520344)
Supplement: Supplementary file 1 — Supporting File: advs73960‐sup‐0001‐SuppMat.docx [file ADVS-13-e20344-s004.docx]

Supporting Information

**Intelligent soft opto-magnetic robot for minimally invasive interventional therapy**

Jingjing Guo^1,2,†,^*, Xiaoyan Guo^1,†^, Jian Wei^3,†^, Miaowen Jiang^4^, Qifa Su^1^, Xian Xia^1^, Zhuozhou Li^1^, Rong Cai^5^, Jianguo Ma^1,2^, Bo Fu^1,2^, Ming Li^4,6^, Jing Zhong^1,2,^*, Lijun Xu^1,^*, Xunming Ji^4,6,7,^*

^1^School of Instrumentation and Optoelectronic Engineering, Beihang University, Beijing 100191, China

^2^Hangzhou International Innovation Institute, Beihang University, Hangzhou, 311115, China

^3^Department of Interventional Radiology, Beijing Friendship Hospital, Capital Medical University, Beijing, 100050, China.

^4^Beijing Institute of Brain Disorders, Capital Medical University, Beijing, 100069, China

^5^School of Engineering Medicine, Beihang University, Beijing, 100191, China

^6^China-America Institute of Neuroscience and and Beijing Institute of Geriatrics, Xuanwu Hospital, Capital Medical University, Beijing 100053, China

^7^Department of Neurology and Neurosurgery, Xuanwu Hospital, Capital Medical University, Beijing 100053, China

*guojj13@buaa.edu.cn; zhongjing@buaa.edu.cn; lijunxu@buaa.edu.cn; jixm@ccmu.edu.cn

^†^These authors contributed equally to this work.

**Supplementary Text**

**Optimization of the iSOM robot design**

The iSOM robot was designed with a soft PVC sheath housing MMFs for delivering sensing and therapeutic light. Except during therapeutic procedures, the robot's tip was not intended to generate heat. Therefore, it was crucial for the robot to maintain tactile perception even at low laser power, such as during navigation. To achieve this, a 6+1 fiber structure was employed to enhance the signal-to-noise ratio of the emission signal, with a central fiber for laser probing and six surrounding fibers for collecting reflected emissions. This design allows tactile sensing with laser power below 10 mW, without inducing noticeable temperature changes at the fiber tip. The leading section of the robot was coated with a ferromagnetic polymer composed of PDMS elastomer with evenly dispersed NdFeB microparticles. The volume fraction of NdFeB was optimized at 30 vol% for optimal magnetic actuation [23]. The length of the coating section was set at about 2 cm to ensure adequate magnetic force for actuation while maintaining the robot's stability and precision in constrained environments.

The GNPs, synthesized in situ within the hemispherical tip, play a key role in enabling pressure detection and photothermal conversion through the excitation of LSPR. The concentration of GNPs was optimized at 0.2% w/v, considering both the pressure sensitivity and photothermal conversion efficiency. The core-shell UCNPs (NaYF_4_:Yb,Er@NaYF_4_), serving as thermal-sensitive molecules, generated multiband visible emissions upon NIR excitation. The substantial spectral overlap between the UCL emissions and the plasmonic resonance of the GNPs enabled simultaneous optical sensing and photothermal ablation under a single NIR excitation. Increasing the concentration of UCNPs was beneficial in enhancing the intensity of UCL emissions. However, excessively high concentrations can hinder the polymerization of PDMS and reduce the mechanical strength of the tip. An optimal concentration of 2% w/v UCNPs was selected for fabrication of the robot tip. Besides, the hemispherical tip was coated with a high-reflective film made of micrometric TiO_2_ particles dispersed in PDMS to minimize light leakage and enhance reflection. With an increase in TiO_2_ concentration, the reflectance of the thin film correspondingly enhanced. A maximum load of 4.5% w/v TiO_2_ was chosen for the coating fabrication. The polymerization of TiO_2_/PDMS samples was monitored by observing the shift in the peak at 2160 cm^−1^, which is associated with the stretching vibration of the Si-H group in the curing agent (Figure S5b). With an TiO_2_ concentration of below 4.5% w/v, the Si–H peaks fully flattened, signaling complete curing of the polymer composites.

**Finite element analysis and modeling**

The deformation of the iSOM robot under magnetic actuation was numerically simulated using a 3D finite element model (FEM) developed in the COMSOL Multiphysics platform. The 'magnetomechanics, no currents' multiphysics interface was used to apply magnetic Cauchy stresses, and the cylindrical magnet was modeled with N42 (Sintered NdFeB) material properties. For the magnetization model, we incorporated the remanent flux density, featuring a recoil permeability of 1.05 and a remanent flux density norm of 1.31 T. For the ferromagnetic polymer composite (30 vol% NdFeB), the magnetization parameter was defined as M=192kA/m, corresponding to a remanent flux density norm of 0.24 T [23]. The magnetization direction of both components is arranged radially. With respect to the solid mechanics interface, the neo-Hookean model was employed to represent the ferromagnetic polymer material behavior, and the compressibility was set as nearly incompressibility. The magnetic-responsive leading section of the robot consists of two parts: a ceramic core and a ferromagnetic polymer coating. The effective Young’s modulus of the leading section can be estimated as following:

$E_{eff}=\left(E_{core}\left(\frac{d}{D}\right)^{4}+E_{coat}\left(1-\left(\frac{d}{D}\right)^{4}\right)\right)$ (1)

where *E_core_* and *E_coat_* represent the Young’s moduli of the ceramic core and the ferromagnetic coating, respectively; d and D denote the core and coating diameter. From Eq. (1), the *E_eff_* was calculated to be about 42 GPa. The rest of the robot's body is primarily composed of a PVC sheath, with an effective Young's modulus of approximately 10 MPa. By adjusting the distance between the iSOM robot and the cylindrical magnet in the simulation, as done in the experiment, we calculated the relationship between the deflection angle of the robot tip and the magnetic field strength. As shown in Figure 5d, the simulated results were in good agreement with the experimental data, which confirmed the validity of the model.

**In vitro cell viability test**

To evaluate the biocompatibility of the iSOM robot, cell viability assays were conducted. SK-N-SH human neuroblastoma cells were seeded on coverslips in 6-well plates, and the robot’s front section (approximately 2 cm in length) was co-cultured with the cells for 24, 48, and 72 hours. Following incubation, the cells were treated with a mixture of Hoechst 33342 and PI (P0137, Beyotime) and imaged using confocal fluorescence microscopy (Dragonfly, Andor). The acquired images were analyzed with ImageJ software. Cell viability was determined by calculating the ratio of blue-stained nuclei (indicating live cells) in the test group relative to the control group. As shown in Figure S13, a high cell viability of above 98% was observed after 72 hours of culture with the robot, confirming its non-toxicity and biocompatibility. These results further validated the iSOM robot's potential for safe use in clinical applications.

**
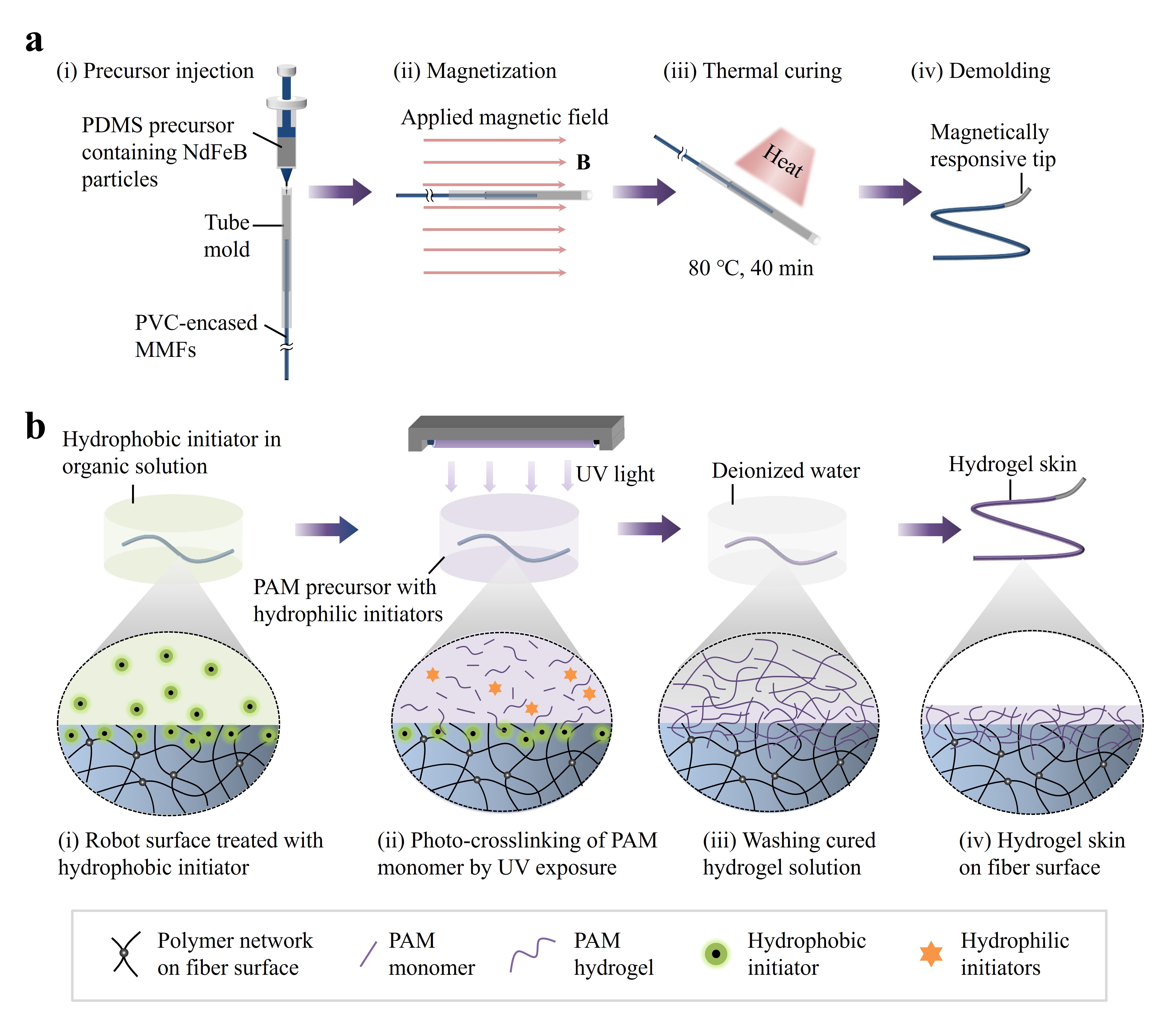
**

**Figure S1.** Fabrication of ferromagnetic polymer and hydrogel coatings for the iSOM Robot. **a,** Fabrication of the ferromagnetic polymer coating. The front section of the PVC-encased MMFs was inserted and positioned at the center of a polyethylene tube mold, followed by injection of a PDMS precursor containing 30 vol% NdFeB particles. The precursor was then magnetized along its axial direction by applying a strong magnetic field. After thermal curing at 80 °C for 5 hours, the coated body was removed from the mold using water pressure. **b**, Fabrication of the hydrogel coating. The robot body was first treated with an ethanol-based solution containing hydrophobic photoinitiators. Afterwards, the treated body was immersed in a PAM precursor that included hydrophilic photoinitiators. After UV curing and washing, a thin hydrogel coating covalently bonded on the robot surface was achieved.


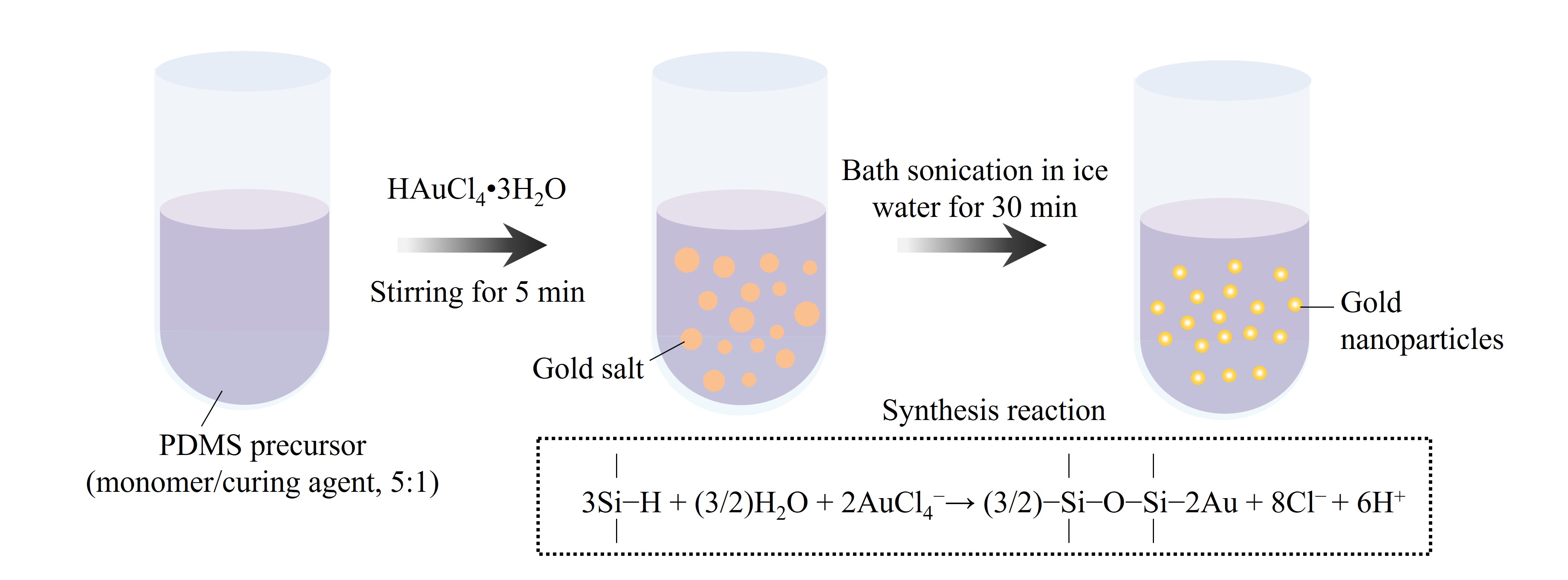


**Figure S2.** Schematic illustration of in-situ synthesis of GNPs in PDMS. The curing agent, abundant in Si-H bonds, acts as a reducing agent to chemically reduce gold salt (HAuCl_4_) into GNPs within the PDMS matrix.


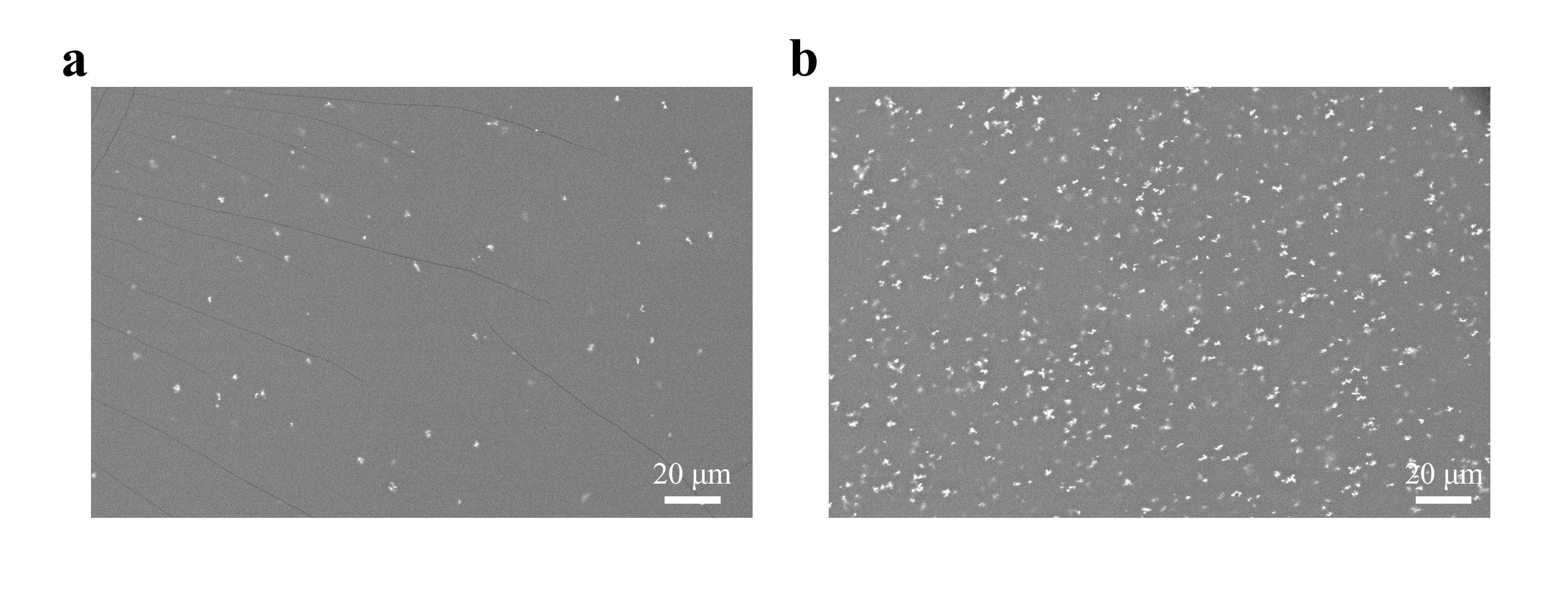


**Figure S3.** Scanning electron microscopy (SEM) images of TiO_2_/PDMS samples with TiO_2_ concentrations of 0.5% w/v (a) and 4.5% w/v (b).


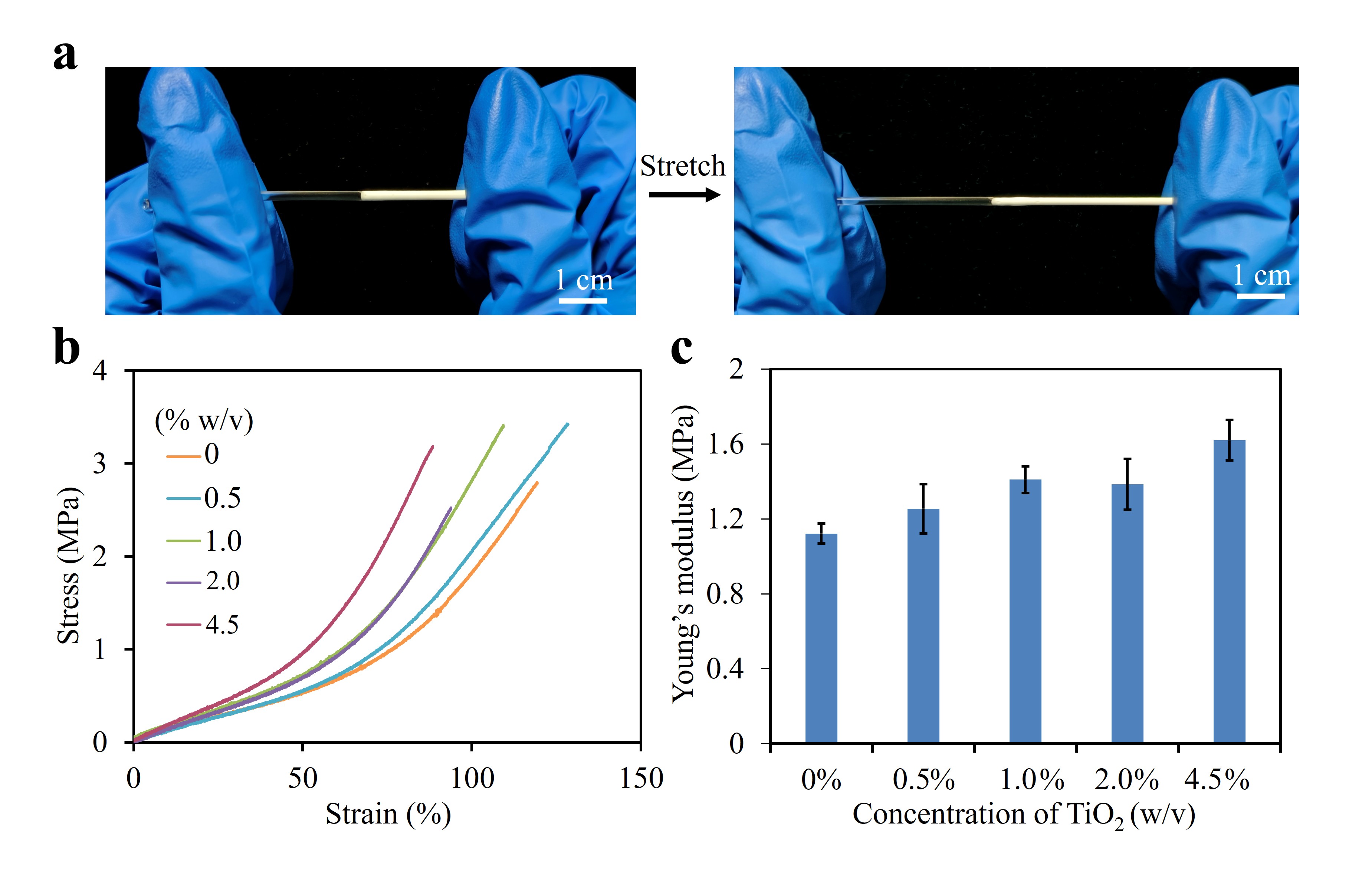


**Figure S4.** Mechanical properties of the TiO_2_/PDMS samples. **a,** Strong adhesion between TiO_2_/PDMS and pure PDMS samples under vigorous deformation, indicating the high robustness of the reflective coating on the tip surface. **b**, Stress-strain curves of TiO_2_/PDMS samples at different concentrations of TiO_2_ particle. **c**, Young’s modulus of TiO_2_/PDMS samples (n=3).


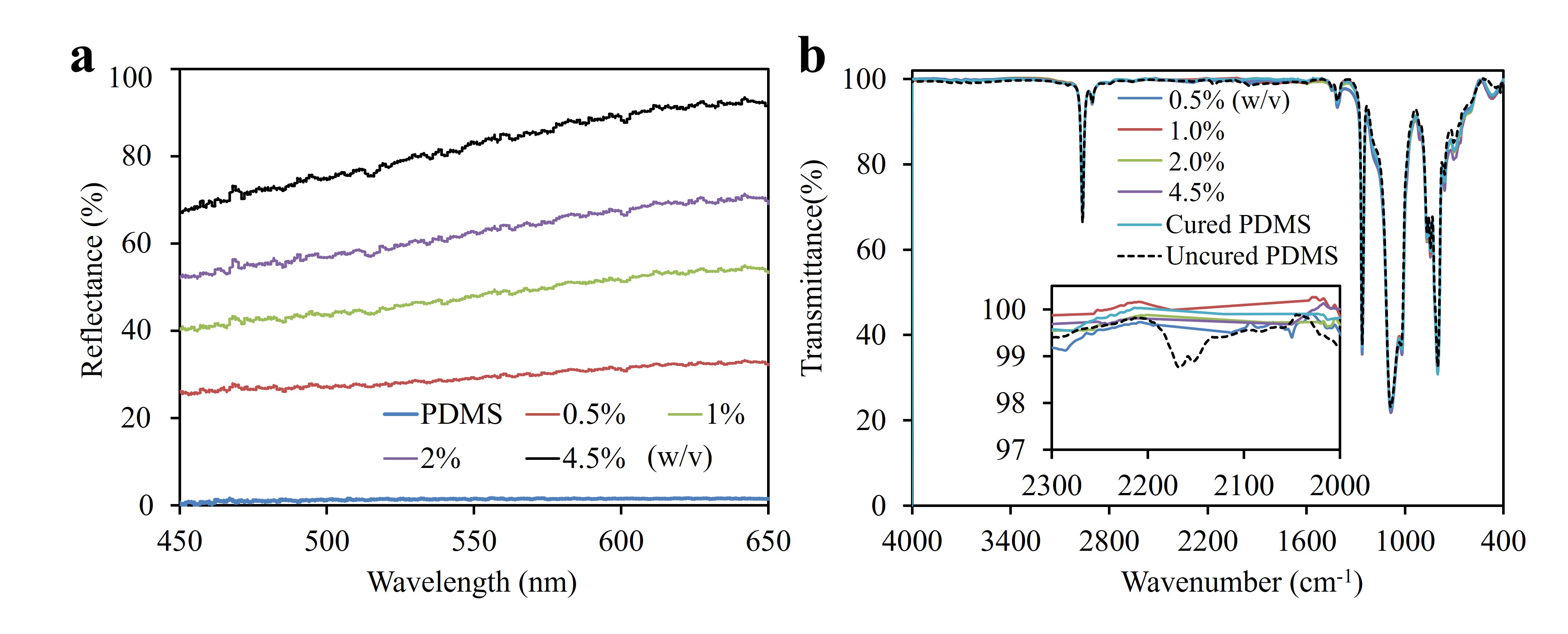


**Figure S5.** **a,** Diffuse reflectance of TiO_2_/PDMS samples at various TiO_2_ concentrations. **b,** FT-IR spectra highlighting the Si-H reactive groups (2160 cm⁻¹) at increasing TiO_2_ concentrations (0, 0.5, 1.0, 2.0, and 4.5% w/v).


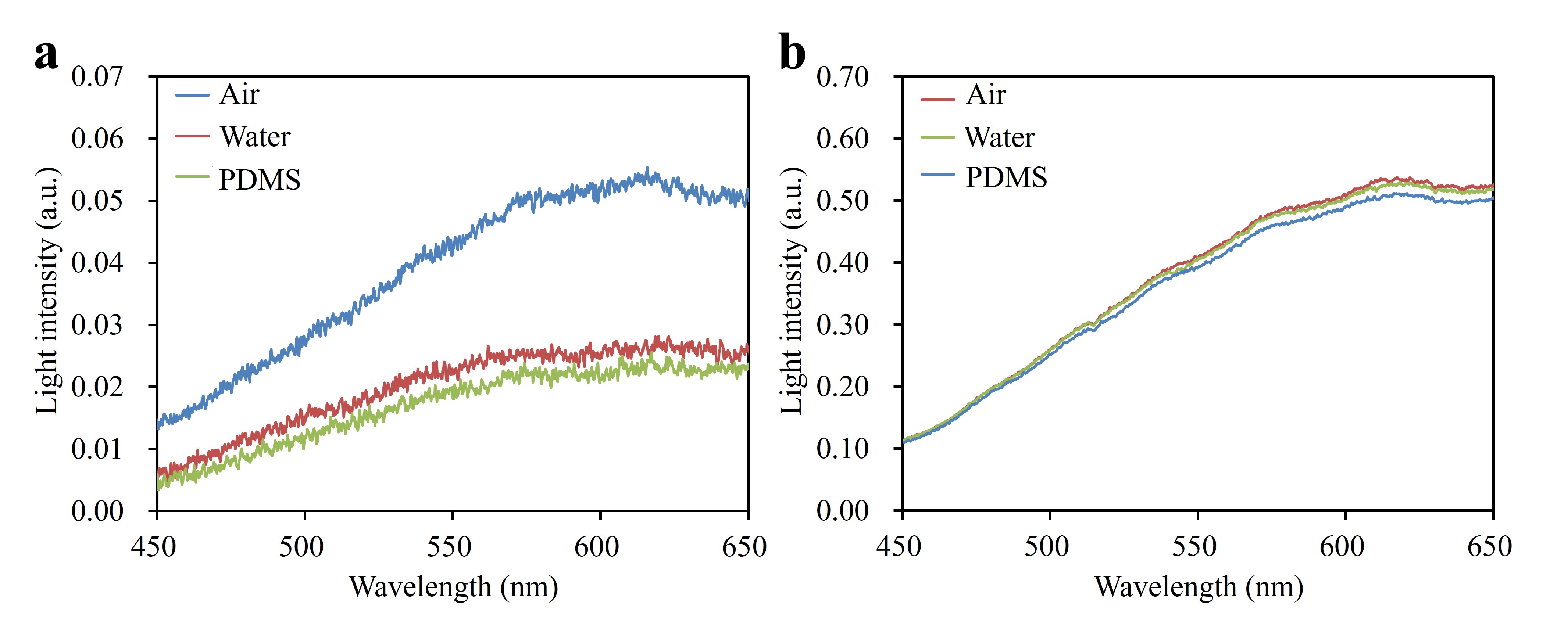


**Figure S6.** Spectra of reflected light from the sensor tip before (a) and after (b) coating with the reflective film under white light illumination. The spectra were measured with the sensor tip immersed in environments with different refractive indices: air (*n*=1), water (*n*=1.33), and PDMS (*n*=1.4). The results indicated a significant enhancement in resistance to environmental interference after applying the reflective coating.

**
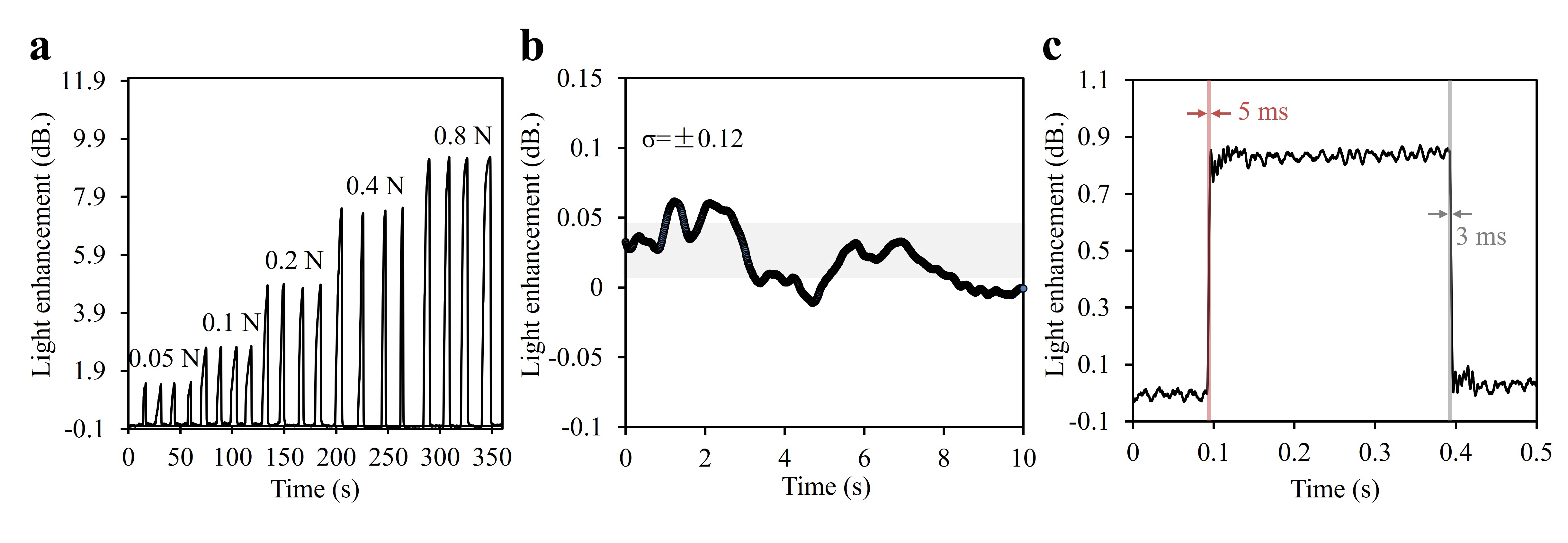
**

**Figure S7.** **a**, Pressure response of the sensor under cyclic loading and unloading at different pressure levels. **b**, Fluctuations in pressure output over time under unloading conditions. **c**, Response and recovery behaviors of the sensor under a quasi-transient step pressure of 0.03 N.


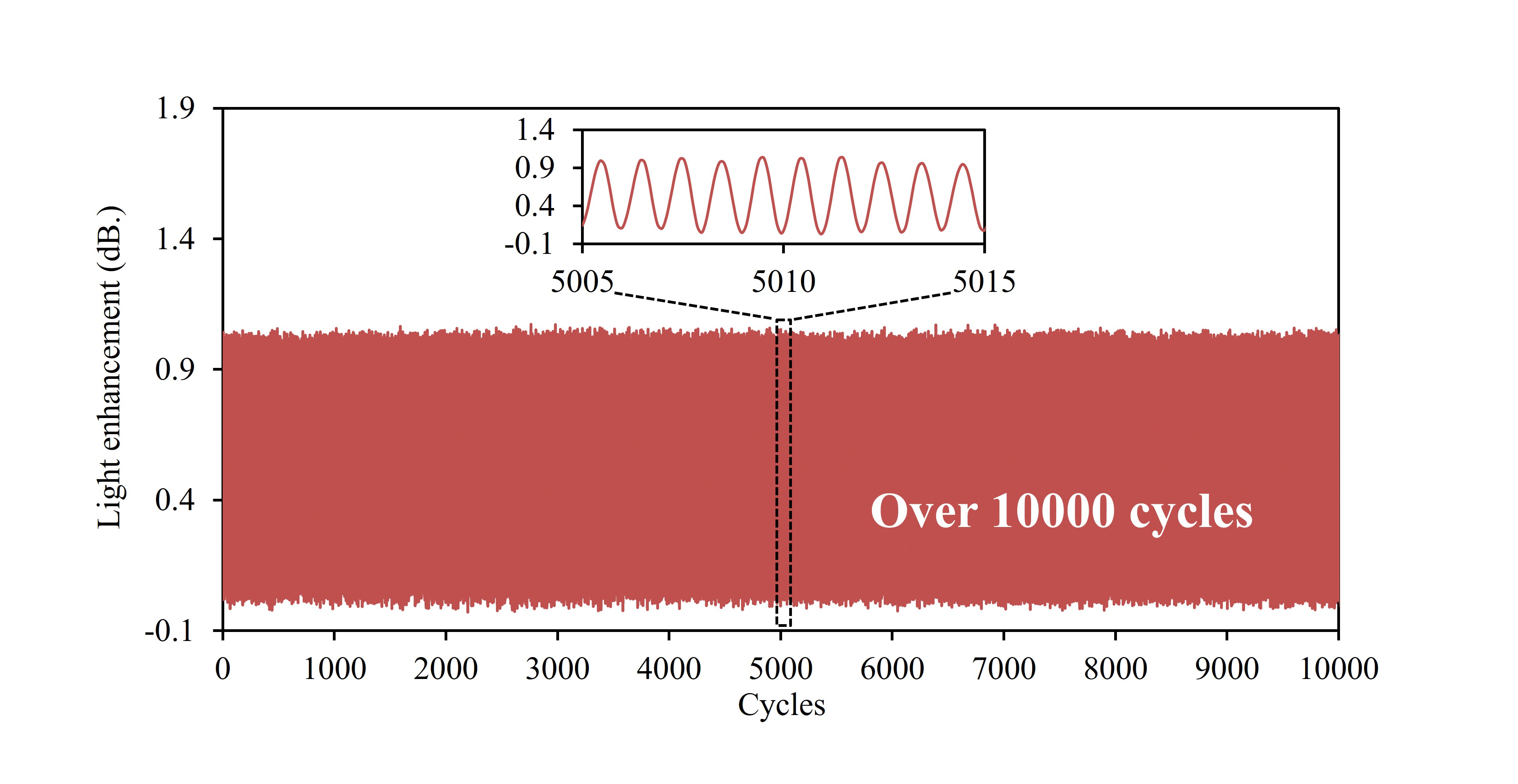


**Figure S8.** Stability and durability test of the sensor under 10000 cycles of pressure loading, demonstrating the sensor's ability to maintain consistent performance and reliability over extended periods of mechanical stress.


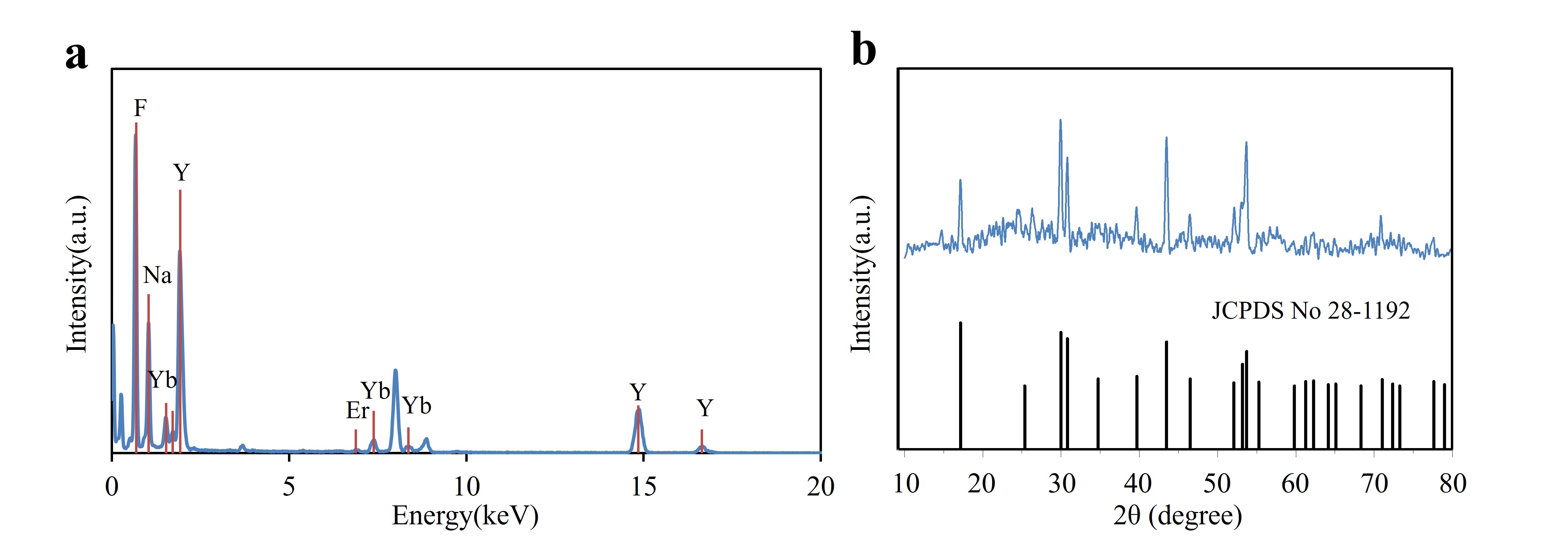


**Figure S9. a**, Energy-Dispersive X-ray (EDX) spectrum of the UCNPs, confirming the presence of elements including Na, Y, F, Er, and Yb. **b**, X-ray powder diffraction patterns of the UCNPs. Compared to the standard (JCPDS 28-1192), the diffraction pattern corresponds to the hexagonal phase.


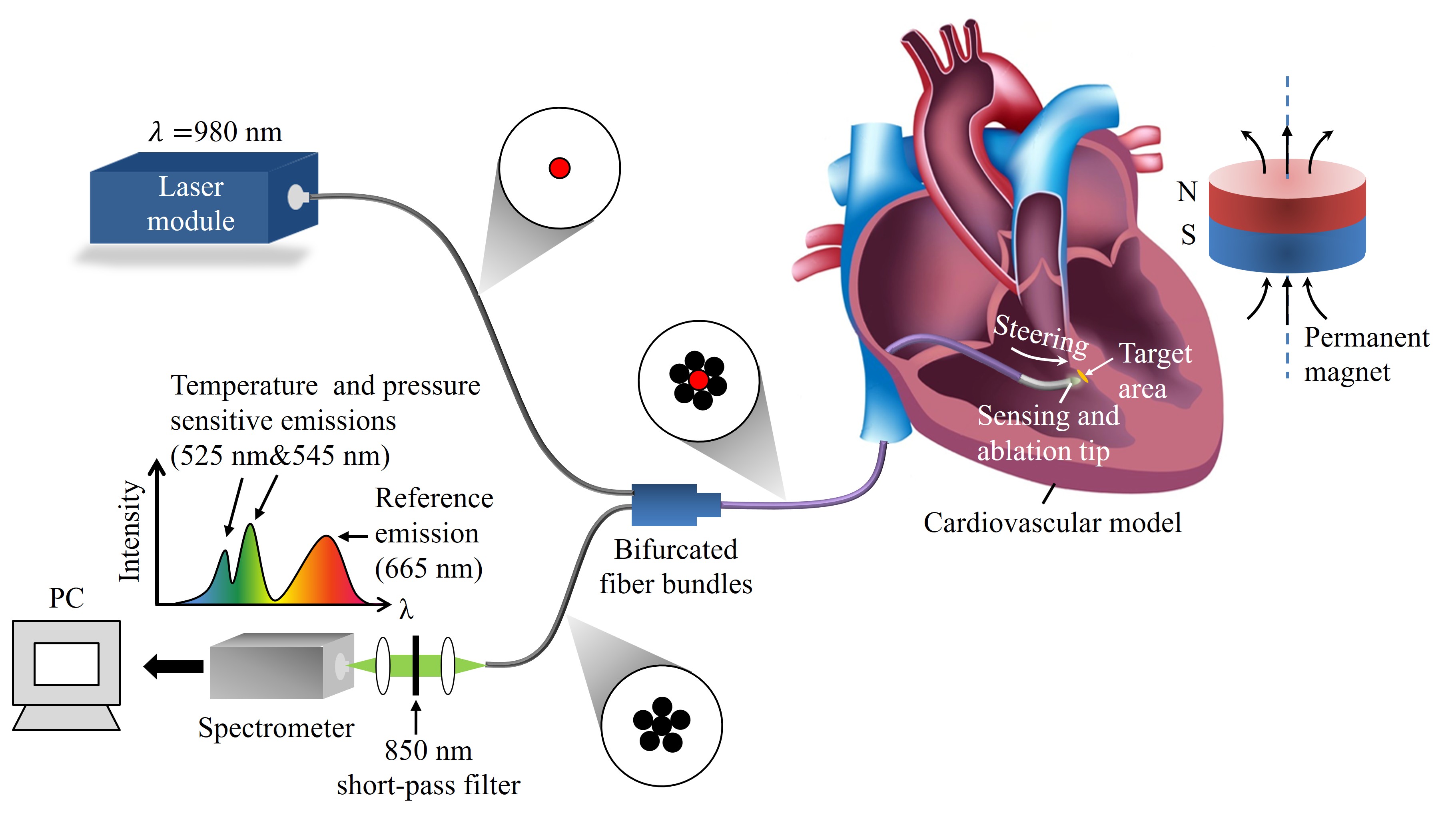


**Figure S10.** Experimental setup for interrogation and actuation of the iSOM robot. A fiber-coupled 980 nm laser was launched into the robot via the central MMF. The reflected emissions were captured by six receiving fibers and directed to a spectrometer for spectral analysis. A short-pass filter with a cutoff wavelength of 850 nm was placed before the spectrometer to suppress the excitation laser. The magnetic field for actuating the robot was generated by a cylindrical permanent magnet, and magnetic steering was achieved by manually adjusting the working distance and orientation of the magnet.


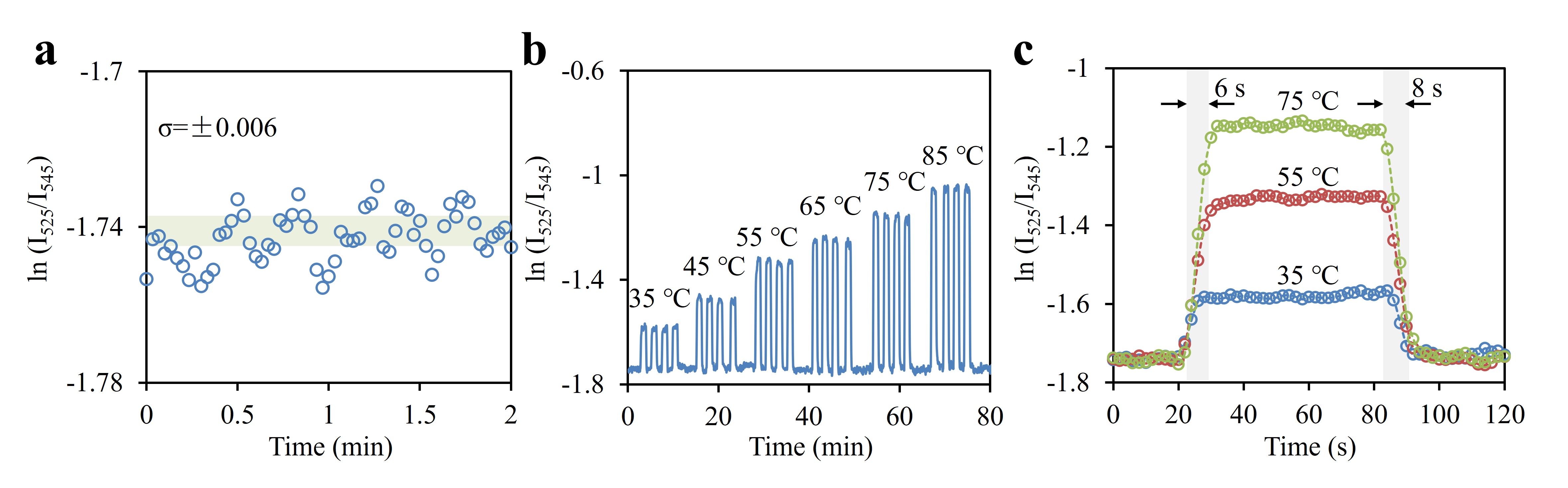


**Figure S11.** **a**, Fluctuations in temperature output over time at a constant temperature of 25 ℃. **b**, Response of the sensor to repetitive loading-unloading cycles of different temperatures. **c**, Temperature response and recovery behavior of the sensor at 35 ℃, 55 ℃, and 75 ℃.

**
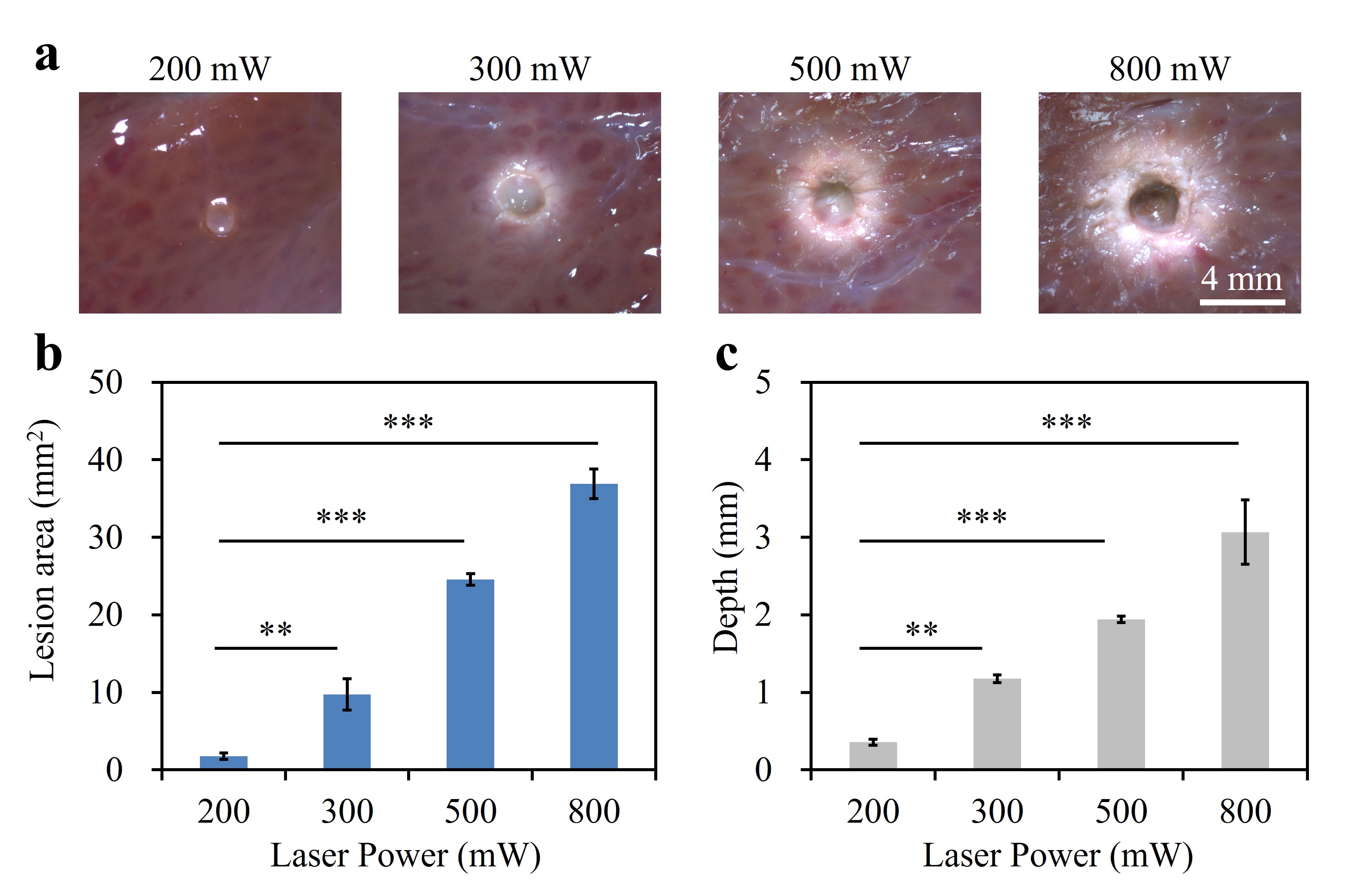
**

**Figure S12.** Quantitative characterization of photothermal ablation in ex vivo liver tissue. **a**, Tissue images showing ablation morphology at different laser powers (ablation duration, 6 min). **b**, Plot of ablation area as a function of laser power (n = 3). **c**, Plot of ablation depth as a function of laser power (n = 3). ** *p* < 0.01, ****p* < 0.001 (student’s t test).

**
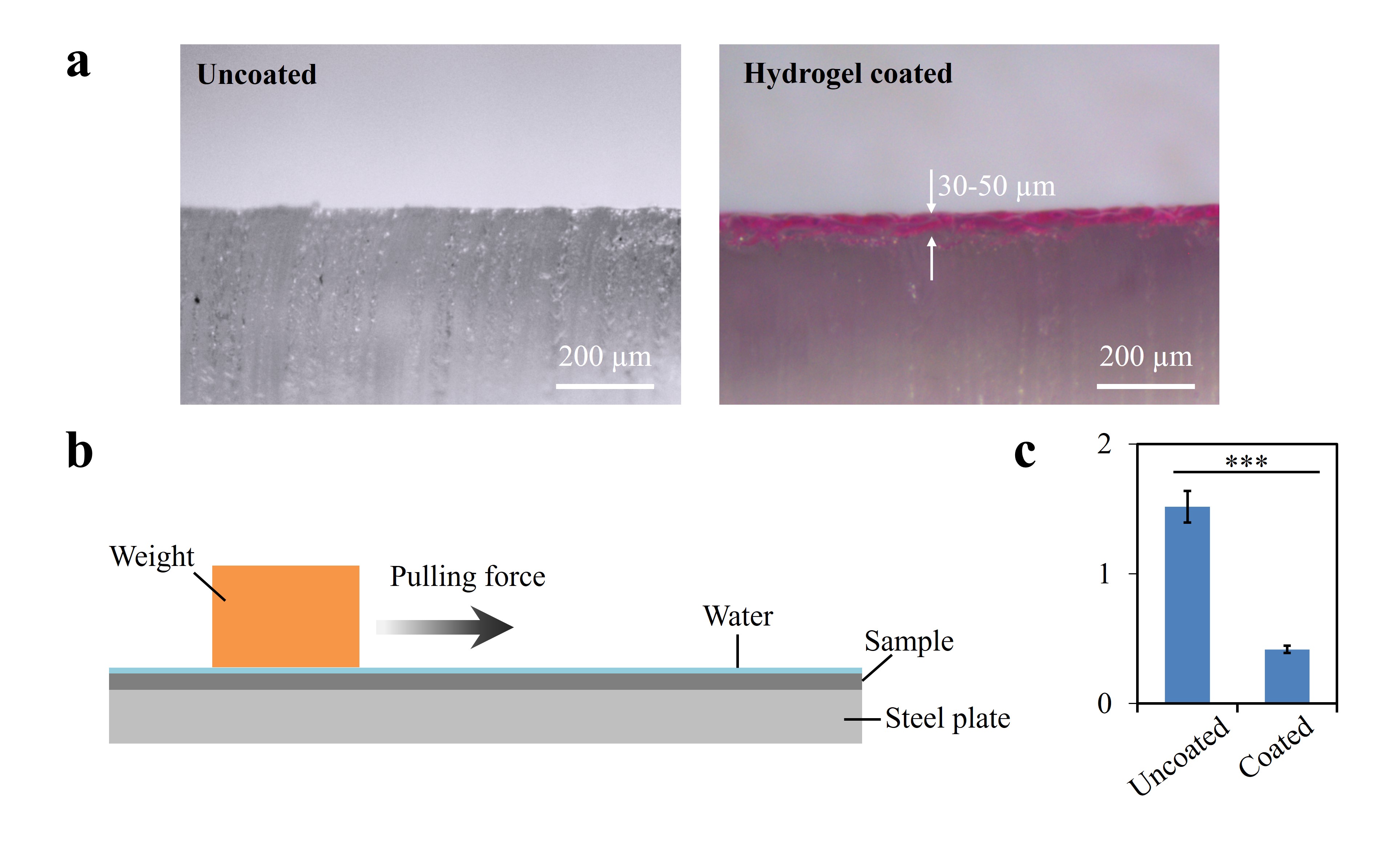
**

**Figure S13**. **a**, Cross-sectional images of PVC samples with/without hydrogel coating, where the hydrogel was stained by red dye. **b**, Schematic of the testing setup for friction coefficient measurement using a friction tester. The PVC sheets were wetted with deionized water, and a 200 g mass was slid across the surface at 100 mm/min. The required sliding force was measured for both coated and uncoated samples, and the friction coefficient was recorded. **c**, Friction coefficients measured before and after applying the hydrogel coating (n=3). ****p* < 0.001 (student’s t test).

**
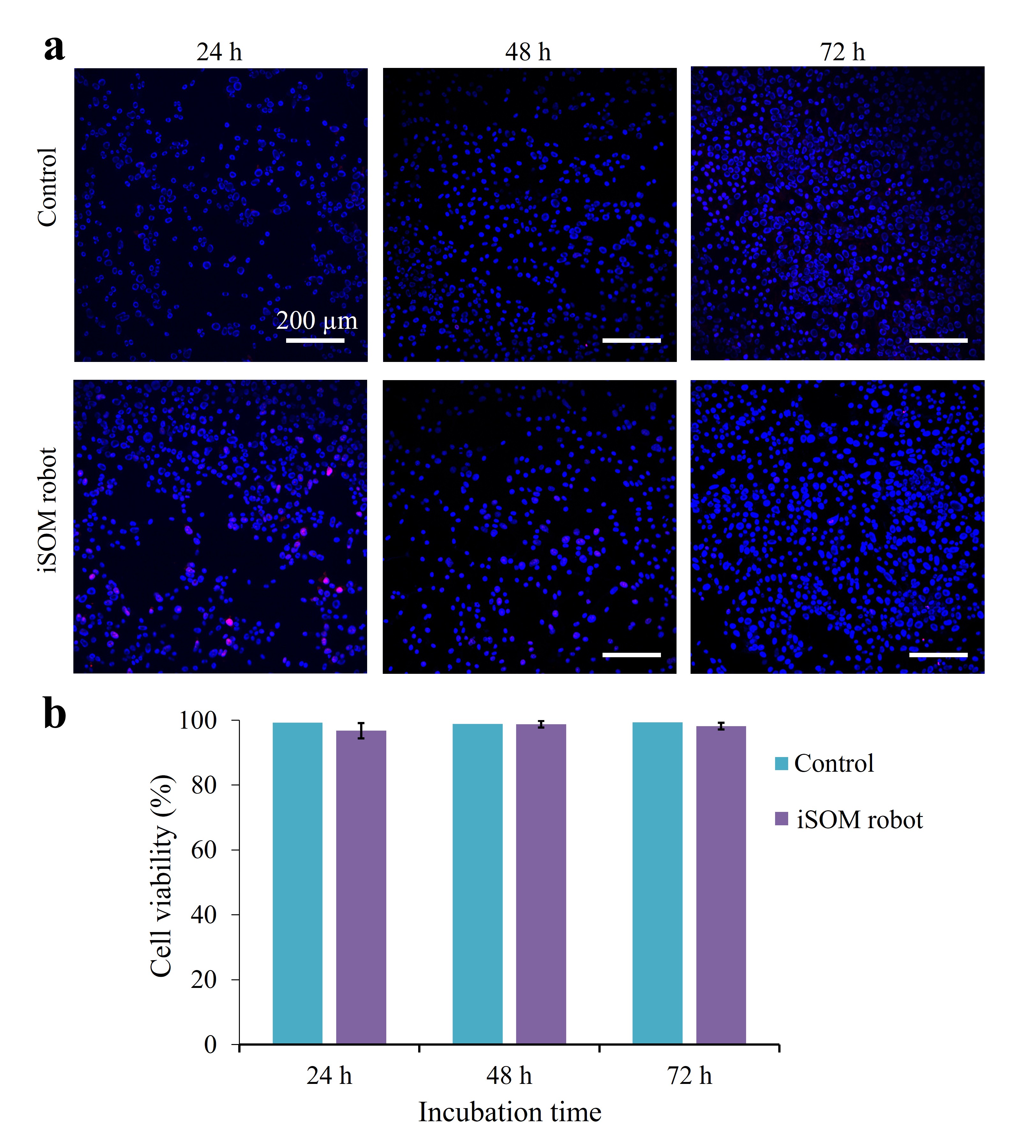
**

**Figure S14.** In vitro cell viability test. **a**, Cell viability assays after co-incubation of SK-N-SH cells with the iSOM robot for 24 h, 48 h, and 72 h. Live cells are shown in blue, and dead cells in red. **b**, Statistical analysis of cell viability.

**
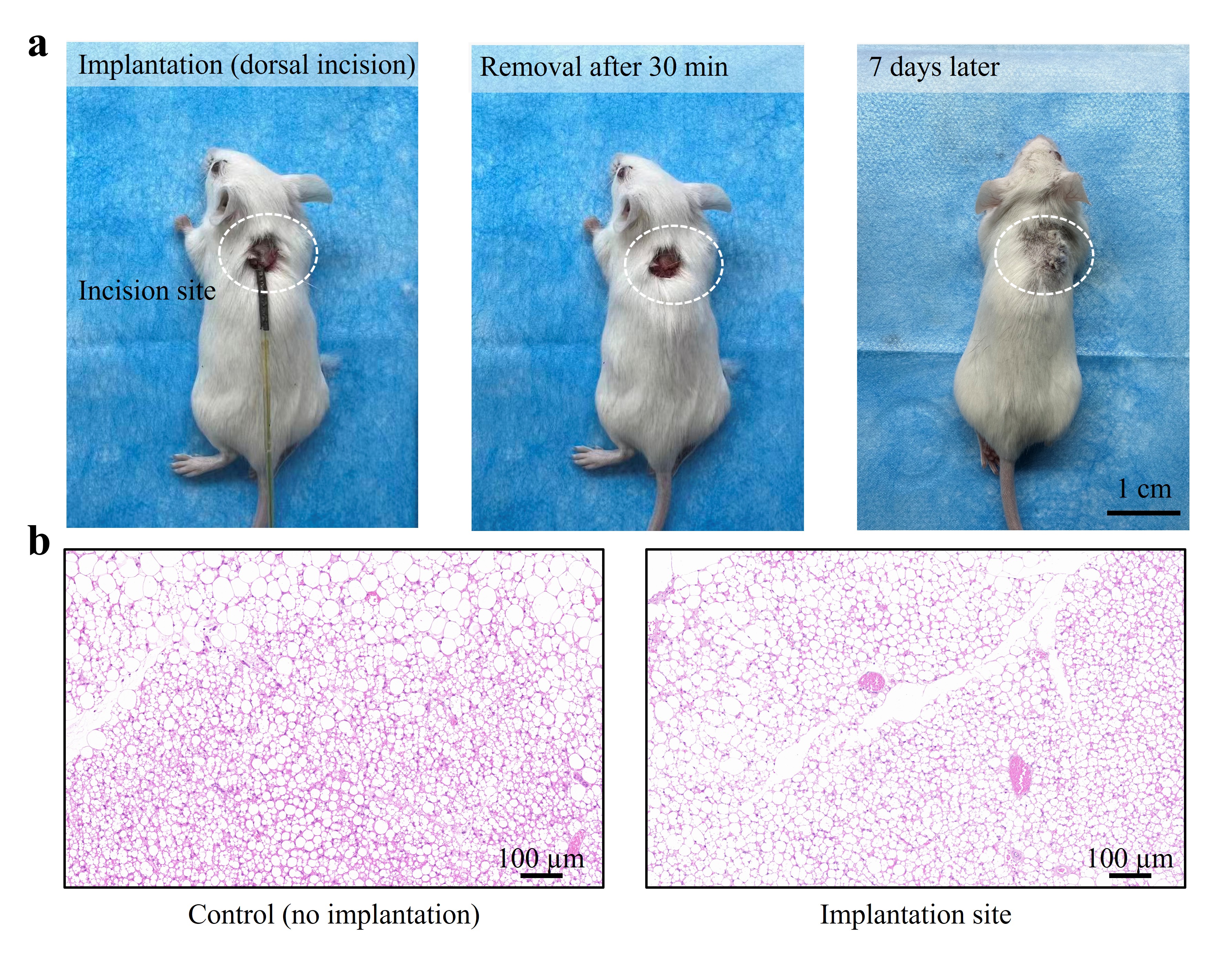
**

**Figure S15.** In vivo biosafety evaluation in mice. **a**, Photographs showing implantation of the iSOM robot through a dorsal incision, removal of the robot after 30 min, and observation of survival and wound healing at 7 days post-procedure. **b**, H&E-stained sections of dorsal tissue from control mice (no implantation) and from mice at the implantation site at the acute time point (24 h post-procedure). Both the control and implantation groups exhibited mild granulocyte and lymphocyte infiltration, with no exacerbation of inflammation or obvious additional pathological abnormalities observed in the implantation group.

**
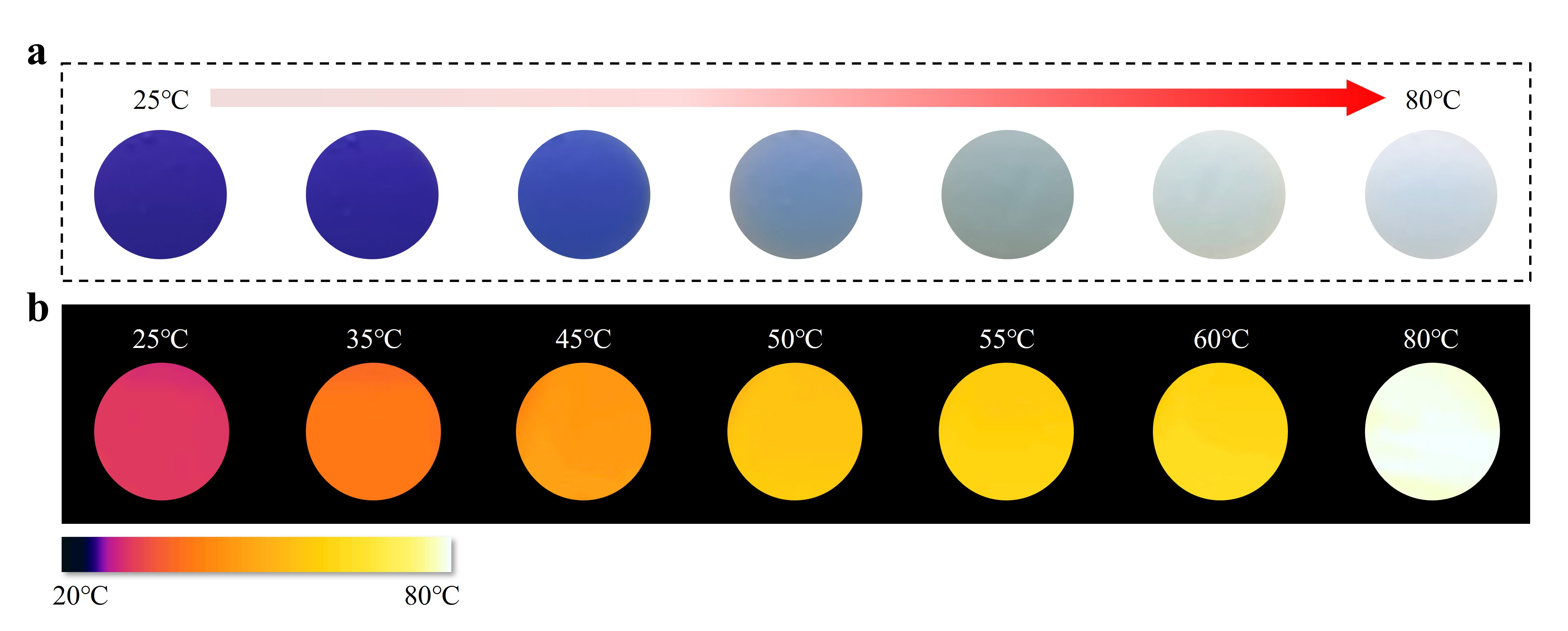
**

**Figure S16.** Temperature-dependent color change of thermochromic ink. **a**, Color change of the ink as it is heated from 25°C to 80°C. **b**, Infrared thermogram of the heated ink. The ink changes from blue to white when the temperature exceeds 55°C.

**
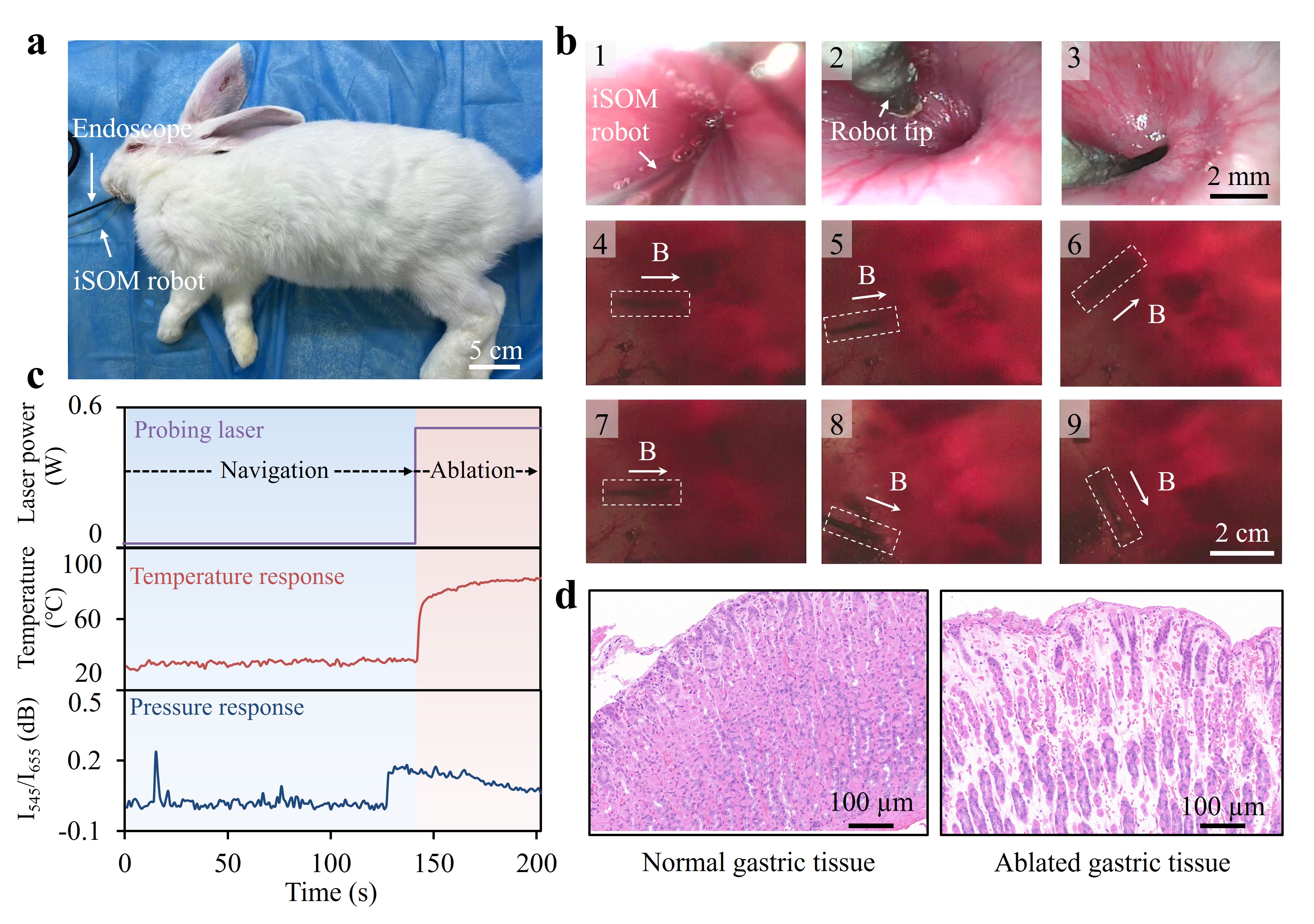
**

**Figure S17.** In vivo gastric interventional validation under endoscopic guidance. **a**, Photograph showing the iSOM robot and the endoscope simultaneously introduced through the oral cavity of the rabbit for gastric intervention. **b**, Endoscopic images illustrating the interventional process: panels 1-3 show advancement through the esophagus into the stomach; panels 4-9 show the iSOM robot magnetically actuated to perform controlled forward and reverse deflections within the gastric cavity under endoscopic visualization. **c**, Real-time pressure and temperature readouts of the robot, along with the probing laser power. **d**, Histological comparison between normal and ablated gastric tissues. The ablated region exhibits histopathological features characteristic of localized photothermal injury, including well-defined thermal necrosis predominantly confined to the mucosal and superficial submucosal layers, accompanied by mild inflammatory cell infiltration and vascular responses.
